# A case of nascent speciation: unique polymorphism of gonophores within hydrozoan *Sarsia lovenii*

**DOI:** 10.1038/s41598-019-52026-7

**Published:** 2019-10-29

**Authors:** Andrey A. Prudkovsky, Irina A. Ekimova, Tatiana V. Neretina

**Affiliations:** 1 Faculty of Biology, Lomonosov Moscow State University, Moscow, Russia; 2 Pertsov White Sea Biological Station, Lomonosov Moscow State University, Moscow, Russia

**Keywords:** Zoology, Biodiversity

## Abstract

Revealing the mechanisms of life cycle changes is critical for understanding the processes driving hydrozoan evolution. Our analysis of mitochondrial (COI, 16S) and nuclear (ITS1 and ITS2) gene fragments resulted in the discovery of unique polymorphism in the life cycle of *Sarsia lovenii* from the White Sea. This polymorphic species exhibits two types of gonophores: hydroids produce both free-swimming medusae and attached medusoids (phenotypic polymorphism). Our phylogenetic analysis revealed the intrinsic genetic structure of *S. lovenii* (genetic polymorphism). Two haplogroups inhabiting the White Sea differ in their reproductive modes. *Haplogroup 1* produces attached medusoids, and *haplogroup 2* produces free-swimming medusae. Our experiments indicated the possibility of free interbreeding between haplogroups that likely is a rare event in the sea. We propose that inter-haplogroup crossing of *S. lovenii* in the White Sea may be limited by discordance in periods of spawning or by spatial differences in habitat of spawning specimens. Our finding can be interpreted as a case of nascent speciation that illustrates the patterns of repeated medusa loss in hydrozoan evolution. Life cycle traits of *S. lovenii* may be useful for elucidating the molecular mechanisms of medusa reduction in hydrozoans.

## Introduction

**Figure 1:**
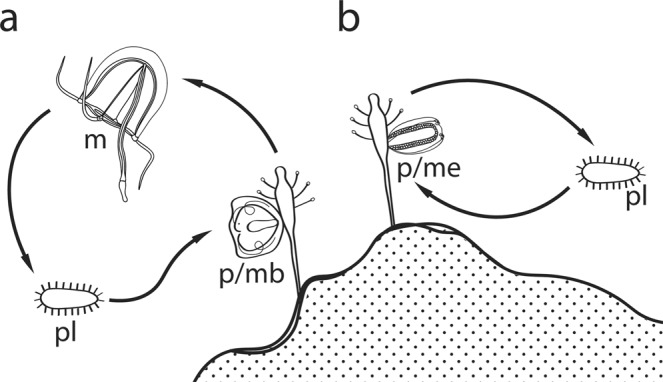
Life cycles of hydrozoans with free swimming medusa (**a**) and reduced gonophore (**b**). Abbreviations: m – medusa stage; pl – planula larva; p/mb – polyp stage with medusa buds; p/me – polyp stage with medusoids.

The evolution of cnidarian taxa is related to changes in life cycle^1^. While Anthozoans and parasitic Endocnidozoans (Myxozoa and Polypodiozoa) lack a medusa stage, in other cnidarian taxa (Hydrozoa, Scyphozoa, Cubozoa) the life cycle typically includes medusa and polyp stages^2,3^. Polyp stage in medusozoan cnidarians produces medusa stage by means of lateral budding in hydrozoans, strobilation in scyphozoans, and metamorphosis of entire polyp in Cubozoans^1^. Recent findings strongly support that Hydrozoa is a sister group to a clade combining Staurozoa (sessile polyp-like jellyfishes) with Cubozoa and Scyphozoa^3^. A specific type of life cycle is one of the main characters used for taxonomical and phylogenetic studies at different hierarchical levels within the medusozoan Cnidaria, especially in hydrozoans. Two hydrozoan groups (Anthoathecata and Leptothecata) have undergone the most frequent and dramatic life history evolution^4^. Within these two taxa, hydroids (benthic polyp stage) produce sexual zooids referred to as gonophores^5^. Gonophores in some hydrozoans develop into medusae that detach from hydroids to swim and feed in the water column (Fig. 1). Free-swimming medusae grow until reaching sexual maturity and spawning gametes. Most hydrozoans, however, lack a fully formed medusa stage and instead produce reduced gonophores (medusoid, sporosac), which are usually retained on hydroids (Fig. 1). Reduced gonophores lack several important morphological characters of medusa such as tentacles, ocelli or even the cavity of the bell^5,6^. The presence of a medusa stage has been asserted to be an ancestral state for Hydrozoa^7^. Despite the apparent advantages of a dispersed feeding pelagic stage, the feeding medusa has been lost at least 70 times across the Hydrozoa^8–10^.

Our knowledge about the medusae reduction phenomenon has been obtained as a result of phylogenetic analysis^9–11^ and the study of gene expression in model species^12–14^. Medusozoan cnidarians with typical medusae possess molecular machinery that enables them to produce medusae. The regulation of genes may be similar in different taxa. For example, indole-containing compounds trigger medusa production across diverse cnidarian species, with the exception of hydrozoans and coronate scyphozoans^15–17^. A molecular mechanism that regulates medusa production was partly suggested for scyphozoan *Aurelia aurita*^15^. Nevertheless, the complete mechanism of medusa production in cnidarians, especially hydrozoan species, is far from comprehensively understood. Although patterns of gene expression and genome mapping have been successfully used for the localization of several regulatory genes, only a limited number of genomes of model cnidarian species have been studied to date^18–21^. Recently, the first draft genome of a hydrozoan jellyfish, *Clytia hemisphaerica*, was reported^12^. The authors studied gene use across life cycle stages and revealed that medusa-specific transcription factors were associated with diverse neurosensory structures. Compared to *Clytia*, the polyp-only hydrozoan *Hydra* has lost many of the medusa-expressed transcription factors. Patterns of gene expression have also been studied in hydractiniid hydrozoans^13,14^. These authors suggested that “*Wnt* pathway elements may play a key role in the loss of the medusa life cycle stage”^13^. At the same time, the comparative study of differential gene expression in two hydractiniid hydrozoans revealed many new candidate genes that may be involved in the evolutionary transitions related to medusa loss or re-evolution^14^. The studied species exhibit the “book-end” phenotypes of gonophore development, with nearly 100 million years of divergence between them^10,14^. It should be useful to compare gene expression in closely related taxa with different types of gonophores.

In our manuscript we study the life cycle of corynid hydrozoan *Sarsia lovenii*^22^, which possibly represent a potential model system for examining the mechanism of the gain or loss of life-history stages in hydrozoans. The hydroid *S. lovenii* was often been confounded with *Sarsia tubulosa*^23^ going back to investigations of XIX century^24–27^. The taxonomy of *Sarsia* hydrozoans is still challenged and the full list of synonyms is presented in most recent review^28^, with discussion on species identity of all described species. Medusae *S. tubulosa* were firstly described by M. Sars^23^ as *Oceania tubulosa*^23^. The species was renamed to *Sarsia tubulosa*^29^ and became the type species of the genus. Hydroids *S. tubulosa* and *S. lovenii* were first described by S.L. Lovén from European waters where colonies with medusa buds were named *Syncoryne sarsii*^30^ (=*S. tubulosa*), and colonies with medusa-like gonophores lacked tentacles and ocelli (medusoids) were identified as *Syncoryne ramosa*. S.L. Lovén believed hydroids with medusoids are identical with the *Stipula ramosa* by M. Sars, 1829^30^. Later M. Sars showed differences in hydroids *Stipula* (*Syncoryne*) *ramosa* (=*Coryne pusilla*) and hydroids described by S.L. Lovén, and renamed the second species as *Syncoryne lovenii*^22^ (=*S. lovenii*). Louis Agassiz (1849) designated similar northwest Atlantic hydrozoans as *Sarsia mirabilis* and supposed the plasticity of its life cycle^24^. He indicated that *Coryne* (*Sarsia*) *mirabilis* hydroids produce medusa in the early spring, but they produce attached medusoids at the end of the budding season in late spring^25^. Consequently, he suggested the same life cycle for European hydrozoans and considered *S. ramosa* (=*S. lovenii*) to likely be only the phase assumed by *S. sarsii* (=*S. tubulosa*) towards the end of the breeding season^26,27^. This suggestion was rejected by C. Hartlaub (1916) and C. Edwards (1978), who clarified the independent status of *S. tubulosa* and *S. lovenii* in European and North American waters^31,32^. According to rearing experiments of C. Edwards (1978), hydrozoan *S. tubulosa* produces only free swimming medusae and hydrozoan *S. lovenii* produces only medusoids^32^. This point of view is also supported in recent reviews^28,33^.

In the present study, we obtained novel data on the *S. lovenii* life cycle by combining morphological and molecular approaches with observations of development and crossing experiments. Our main goal was to validate the species identity of attached medusoids and free-swimming medusae of *Sarsia* in the White Sea and to verify a hypothesis about *S. lovenii* life cycle polymorphism.

## Results

### Molecular analysis

#### Phylogenetic trees

**Figure 2:**
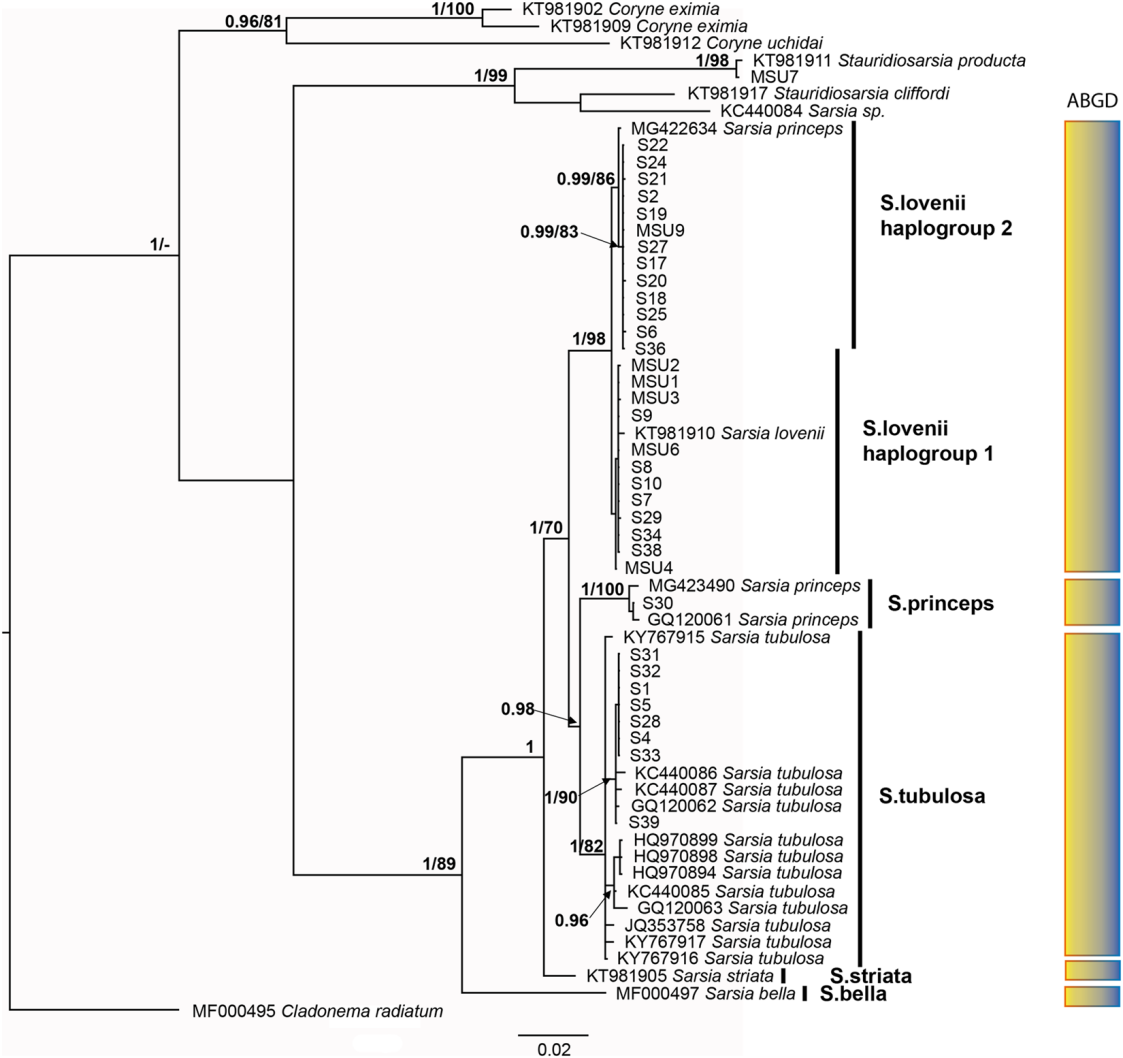
Bayesian phylogenetic hypothesis based on the combined mitochondrial and nuclear dataset (COI-16S-ITS). The first numbers on branches represent posterior probabilities from Bayesian inference, and the second numbers indicate bootstrap values from maximum likelihood (1000 pseudoreplicates). The results of ABGD analysis are presented in the right column.

We obtained 122 new sequences of corynid hydrozoans from the White Sea: 35 for 16S, 38 for COI and 49 for ITS (Supplementary Information Table S1, Figs S1, S2). Substitution saturation plots revealed no saturation for any gene. All single-gene trees (Supplementary Information Fig. S3) and the concatenated tree (Fig. 2) produced congruent topologies, and differences between them were related to differences in taxon sampling. Node support was lower in the case of single-gene trees with several polytomies, while the concatenated dataset provided well-resolved and supported relationships.

The genus *Sarsia* was recovered as paraphyletic due to the nesting of a *Sarsia* sp. specimen (acc. number KC440084) within *Stauridiosarsia* species, which was most likely the result of misidentification. Following the exclusion of this specimen, the genus *Sarsia* was recovered as monophyletic (PP = 1, ML = 89). Within this genus, five monophyletic species-level groups were recovered: *S. tubulosa, S. principes, S. lovenii, S. striata*, and *S. bella*. The sixth *Sarsia* species, *S. apicula*, was only discerned on the 16S-tree since its COI sequence was not available (Supplementary Information Fig. S3). The 16S dataset also provided a separated clade with two specimens showing identical sequences (*S. tubulosa* - EU876548 and *S. princeps* - EU876549), which were placed outside of traditional species lineages. We excluded those sequences from the analyses because of their ambiguity (see discussion chapter). Several specimens of medusae from the White Sea formed a clade with *S. tubulosa* from GenBank (PP = 1; ML = 82). Other medusa specimens were nested with *S. lovenii* specimens from GenBank and the specimens from the White Sea that possessed medusoids (PP = 1; ML = 98). Additionally, a medusa specimen from Canadian waters (MG422634) that was identified as *S. princeps* was grouped with the *S. lovenii* clade.

#### Genetic diversity in Sarsia tubulosa and S. lovenii

The uncorrected *p-*distance between *S. lovenii* and *S. tubulosa* clades ranged from 3.9% to 5.4% in the COI dataset. The interspecific distances were less evident in the 16S dataset, and ranged from 0.7% to 2.1% (Supplementary Information Table S2). Maximal intraspecies distances (1.4%) exceed minimal interspecies values for the 16S dataset. Uncorrected *p-*distances within the *S. tubulosa* clade using the COI dataset ranged from 0 to 1.7%. Uncorrected *P-distance* between specimens from two subclades of *S. lovenii* using the COI dataset ranged from 1.2% to 1.7%.

**Figure 3:**
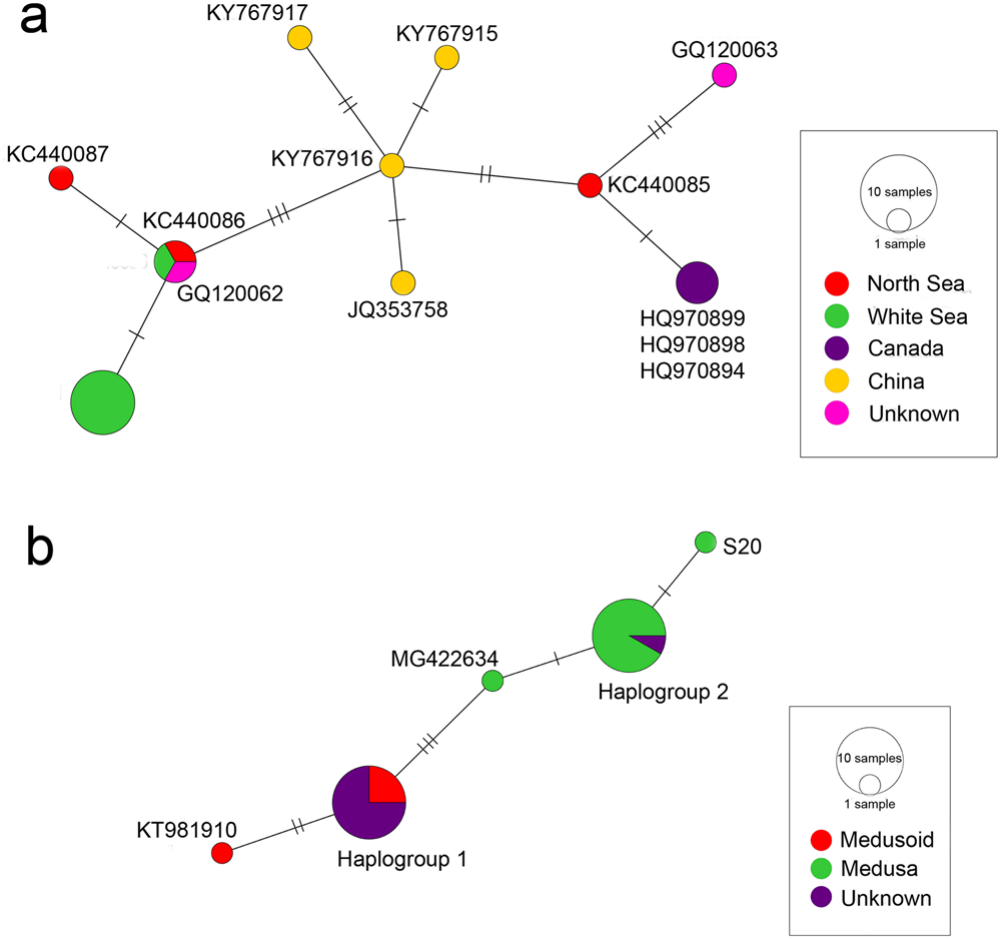
COI haplotype networks of *Sarsia tubulosa* and *Sarsia lovenii* produced via the TSC method in PopART: (**a**) *Sarsia tubulosa;* the geographic region where each haplotype was found is colour coded. (**b**) *Sarsia lovenii*; the gonophore type of each haplotype is colour coded. The relative size of circles is proportional to the number of sequences of that same haplotype.

In addition, we revealed apparent intraspecific genetic structure in *Sarsia tubulosa* and *S. lovenii* (Figs 2 and 3). The haplotype network of *S. tubulosa* contained 20 specimens representing 10 haplotypes from the White Sea, North Sea, and coastal waters of Canada and China. The distribution of *S. tubulosa* haplotypes correlated with the geographic site from which the isolates were collected. Two haplotypes from the White Sea were separated by one mutational step. The first highly frequent haplotype was represented by seven specimens, and the second low-frequency haplotype was found in one specimen from the White Sea as well as two specimens from the North Sea and an unknown locality. Two haplotypes from the North Sea were separated by one mutational step. A third haplotype from the North Sea was separated from coexisting specimens by five nucleotide substitutions, but it was closely related to the Canadian haplotype (one mutational step). Four haplotypes from Chinese waters were interconnected by one or two mutation steps. These haplotypes were separated by 2–3 substitutions from neighbouring haplotypes from other locations.

The haplotype network of *S. lovenii* was represented by five haplotypes, mostly including specimens from the White Sea. There were two highly frequent haplotypes, each was detected in 12 specimens. The first frequent haplotype from the White Sea and a haplotype from the North Sea formed *haplogroup 1*; this haplogroup formed a monophyletic clade on the concatenated tree (PP = 0.75, ML = 58) and included several polyp specimens and specimens with medusoids. The second frequent haplotype from the White Sea was interconnected by one mutational step with a rare haplotype from the White Sea and a haplotype from Canadian waters. These three haplotypes formed *haplogroup 2*, which mostly included medusa specimens and one polyp specimen with an unknown type of gonophore. Specimens from *haplogroup 2* formed a monophyletic clade on the concatenated tree (PP = 0.99, ML = 86). The two haplogroups were separated by three nucleotide substitutions (Fig. 3b).

We also analysed phylogenetically important nucleotide substitutions in *Sarsia lovenii* specimens from two haplogroups using full COI and ITS datasets (Table 1). We found seven substitutions in the COI dataset and three substitutions in the ITS dataset. The differences in the nucleotide substitutions in COI and ITS datasets were congruent apart from one exception. Polyp specimen “MSU4” nested within *haplogroup 2* according to ITS fragment but grouped with *haplogroup 1* according to its COI fragment (Table 1).

**Table 1:** Phylogenetically important nucleotide substitutions (COI and ITS) of specimens *Sarsia lovenii* of two haplogroups (Fig. 2) and offspring specimens in crossing experiments.

Specimens *Sarsia lovenii*	Nucleotide positions in datasets							
	ITS		COI					
	136–140	534	26	356	371	417–419	558	602
Haplogroup 1 (medusoid)	CGC––	C	T	C	A	TTA	T	C
Haplogroup 2 (medusa)	CGCGC	T	C	T	G	CTG	C	T
Offsprings (S43, S47–S49) in experiment 1 (Female-medusa × Male-medusoid)	CGC––	**R**	N	T	G	CTG	C	T
Offsprings (S44–S46, S51) in experiment 2 (Female-medusoid × Male-medusa)	CGC––	**R**	N	C	A	TTA	T	C
MSU4	CGC––	T	T	C	A	TTA	T	C

Heterozygous nucleotides abbreviated with bold type.

#### Species delimitation

The ABGD analysis using the default parameters retrieved eight partitions. The first one revealed signals of oversplitting, and the others revealed initial partitions with five groups (P = 0.0028–0.0077) (Fig. 2; Supplementary Information Fig. S4). The splitting of all specimens into five groups is congruent with the traditional species taxonomy (*S. tubulosa, S. lovenii, S. princeps, S. striata*, *S. bella*).

### Crossing experiments

**Figure 4:**
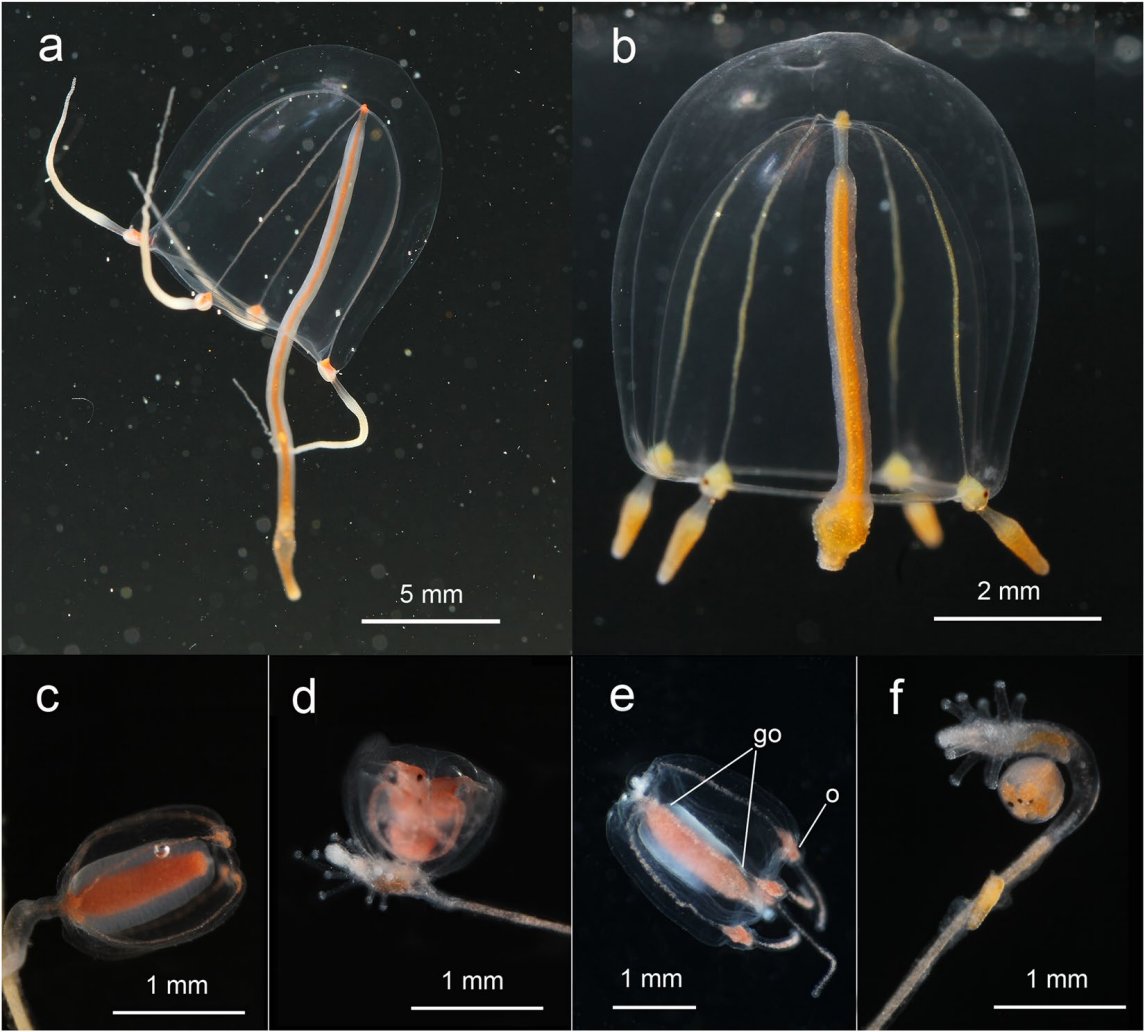
Photographs of *Sarsia lovenii* and *Sarsia tubulosa* specimens: (**a**) Medusa specimen (s21) of *S. lovenii*, magnification x4.1. (**b**) Medusa specimen (s28) of *S. tubulosa*, magnification x12.4. (**c**) Polyp specimens of *S. lovenii* with medusoid (s34), magnification x19.6. (**d**) Polyp specimens of *S. lovenii* with medusa bud (MSU9), magnification x19.6. (**e**) Medusa specimen (S51) of *S. lovenii* produced by experimental colony (result of interbreeding between medusoid female and medusa male), magnification x19.6. (**f**) Polyp specimen (s39) of *S. tubulosa* with medusa bud, magnification x19.6. Abbreviations: go – gonad; o – ocellus.

Interbreeding experiments in which female gametes from a *Sarsia lovenii* medusoid were crossed with male gametes from an *S. lovenii* medusa or vice versa resulted in successful fertilization and ultimately in viable colonies (Supplementary Information Fig. S5). Eggs did not develop in the negative control without males. The results of the molecular analysis supported the success of our crossing experiments: the mitochondrial COI fragment identified in offspring specimens showed the same haplogroup as in the maternal specimens, but the nuclear ITS fragment of the offspring specimens contained a heterozygous nucleotide position (Table 1). The offspring colonies produced medusae buds at water temperatures of 0–2 °C. The gonophores had four radial canals, four tentacle’s bases with ocelli, four tentacles and a manubrium which reached the rim of the bell (Fig. 4; Supplement Information Fig. S2: specimens S49 and S51). Gonads were formed while gonophores were still attached to polyps (Fig. 4; Supplementary Information Fig. S2: specimen S51). In our experiments all gonophores were males. The gonads were located at the basal 2/3 of manubrium keeping free distal tapered part of manubrium. We found motile spermatozoids in gonads of several examined gonophores. Sometimes medusae with ripe gonads separated from the parental colony and swam near the bottom of a bowl. Free swimming medusae caught nauplii of *Artemia* by tentacles but did not ingest them. They reached a size of 2,5 mm. Medusae survived up to two weeks in a bowl at a water temperature of 5 °C.

### Morphology of Sarsia lovenii medusae

**Figure 5:**
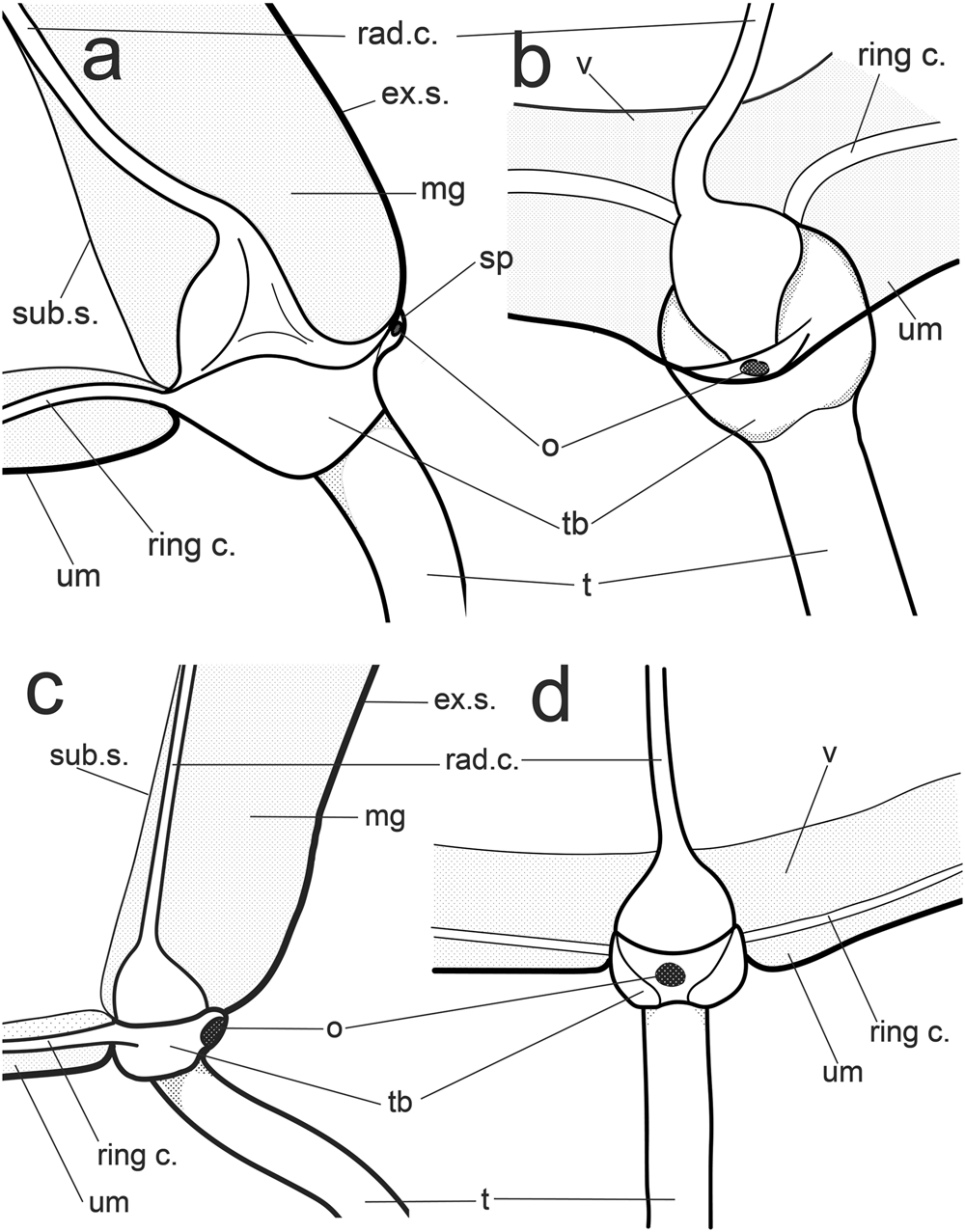
Shape of the medusa tentacle bulb in side view (**a,c**) and front view (**b,d**): (**a,b**) *Sarsia lovenii*; (**c,d**) *Sarsia tubulosa*. Abbreviations: ex.s. – exumbreal surface; mg – mesoglea; o – ocellus; rad.c. – radial canal; ring c. – ring canal; sp – spur; sub.s. – subumbrellar surface; t – tentacle; tb – tentacular base; um – umbrella margin; v – velum.

The morphological analysis indicated some differences in the morphology of *Sarsia* medusae in the White Sea (Figs 4 and 5) and was strongly congruent with our molecular results. Since the medusa stage has not yet been described for *S. lovenii*, we provide a detailed description of this stage for the first time using the description of *S. tubulosa* medusae by P. Schuchert^28^ as a template.

Adult medusae of *S. lovenii* exhibit bells of approximately 7–10 mm (up to 16 mm) in height, slightly higher than wide, bell-shaped, with interradial exumbrellar furrows. The manubrium may be very long in living medusae, 2–3 times as long as the bell, but in preserved specimens, the manubrium is strongly contracted and often shorter than the bell. An apical chamber (knob) of variable shape is usually present, with or without an apical canal (Supplementary Information Fig. S6). The manubrium is composed of a long, broad, proximal part and a spindle-shaped swelling at the distal end (stomach). The stomach is inflated when it is filled with food. The gonads usually encircle most of the manubrium along the proximal part and end distally at the beginning of the stomach. Radial canals enter the gastrodermal chambers of bulbs on the abaxial side and pass through the mesoglea. The ring canal forms a small curve in the vertical plane before entering the bulb. Tentacle bulbs are large with an abaxial black ocellus, which is located on the arched distal process of the bulb (spur) (Fig. 5). The colour of bulb and apical knob is usually orange. The main differences between *S. lovenii* and *S. tubulosa* medusae are summarized in Table 2.

**Table 2:** The morphological differences between *Sarsia lovenii* and *Sarsia tubulosa* medusa.

Characters	*Sarsia tubulosa*	*Sarsia lovenii*
Bell size in adult specimens (mm)	3.5–6	7–10 (up to 16)
Colour of tentacle bulbs and apical knob	greenish and light orange	light orange
Shape of tentacle bulbs	round	with spur (distal process of bulb)
Position of gonad over manubrium	distal 2/3 of manubrium	most part of manubrium

### Production of gonophores by hydroids *S. lovenii* and *S. tubulosa* in the sea and in laboratory, and spawning period in dependence to temperature

**Figure 6:**
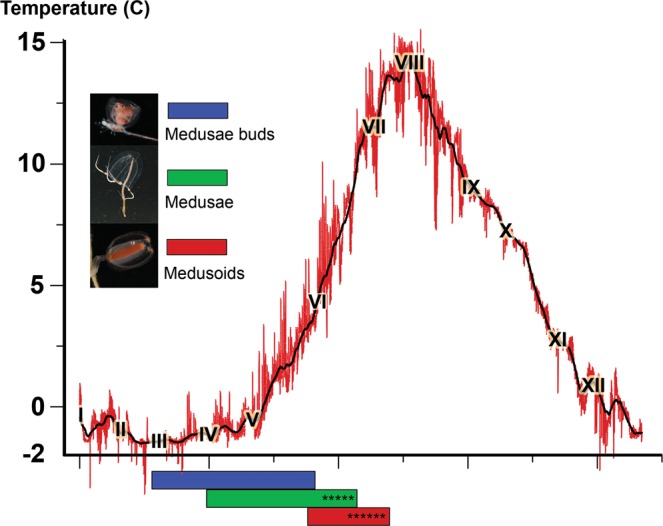
Scheme of the periodicity of gonophore production by *Sarsia lovenii* throughout the year in the White Sea in comparison to surface temperatures of the sea from 2017. Key: Red line, measured temperature data; black line, 7-day averaged temperature data; months are abbreviated with Latin numerals; asterisks indicate spawning period in medusae and medusoids.

Medusae of *S. tubulosa* were found mainly in June and July in the “Saline lake at the Green Cape” that is partly isolated from the sea by low rapids and has independent temperature dynamic (Supplementary Information Fig. S1; Table S1). The temperature of the sea surface at this locality reached 16 °C in June 2016. Also several *S. tubulosa* medusae were collected at other localities in July and August. We have not found any *S. tubulosa* colonies in the sea, but a colony with medusa bud (S39) was collected from an aquarium. *Sarsia lovenii* polyps with a medusae buds were collected in April (S11) (Supplement Information Table S1, Fig. S1). Sporadic field observations in 2012–2019 indicated presence of these polyps at “Eremeevskie rapids” site from the end of February till the end of May when the temperature of the sea surface increase from negative values to 3–5 °C (Fig. 6). Hydroids of *S. lovenii* produced medusae buds in the laboratory at the temperature range of 0–6 °C (Supplement Information Fig. S7). *Sarsia lovenii* medusae were collected near the White Sea Biological Station from April till June (Supplement Information Fig. S1, Table S1). Medusae collected in June 2018–2019 at a temperature 5–10 °C were ripe and began to spawn being placed in aquaria. Polyps of *S. lovenii* with medusoid (S29, S35, S38) were collected at “Eremeevskie rapids” site in June (Supplement Information Table S1, Fig. S1). According to sporadic field observations in 2012–2019 these polyps can be collected at this locality from the end of May till July, when the temperature of the sea surface increase from 5 °C to 10–15 °C (Fig. 6). Hydroids of *S. lovenii* produced medusoids in laboratory at a temperature 2–5 °C and 4–6 °C (Supplement Information Fig. S7). Medusoids collected from the middle of June till July in 2018–2019 at a temperature 10–15 °C were ripe and began to spawn being placed in aquaria.

As a result of molecular analysis (Supplementary Information Table S1) and long-term sporadic field observations of medusae and hydroids, we propose a scheme of the *S. lovenii* life cycle in the White Sea (Fig. 6). Hydroids of *haplogroup 2* produce medusae in spring (from March to May) at water temperatures of −1.5–5 °C. Medusae appear in plankton beginning in April and decrease in numbers by the end of June. They spawn in June at a water temperature of about 10 °C. Hydroids of *haplogroup 1* produce medusoids from May through July at water temperatures of 5–15 °C. Medusoids spawn in June and July.

## Discussion

Gonophores characterized by reduced medusae traits are known in the most families of Anthoathecata and Leptothecata^4^. The extent of reduction varies from an almost developed medusa with subumbrellar cavity and radial canals named eumedusoid to more regressive gonophore without radial canals and sometimes subumbrellar cavity (cryptomedusoid, heteromedusoid), or even to the most regressed gonophore named styloid or sporosac that lacks any trace of medusa-like characters^4–6^. Evolutionary mechanisms of medusae reduction have not been revealed yet and taxonomic significance of such morphological regressions is ambiguous. Our results suggest the existence of two gonophore types within the species *Sarsia lovenii*. Medusa-like gonophores without tentacles and ocelli (eumedusoids) are a common type of gonophore that is well known in *S. lovenii, w*hile we demonstrated for the first time that hydroids produce free-swimming medusae as well.

The systematics of the corynid hydrozoans are undergoing revaluation^28,34–36^. Only a few species described in the XIX century and the first part of the XX century are valid to date^28^. The medusae of *Sarsia* lack significant distinguishing features to differentiate one species from another. It was only after hydrozoan scientists started to work with living specimens that the quality of species identification became more reliable^28^. Nevertheless, their methods were insufficient in some intricate cases. We now have powerful molecular tools that make identification more reliable, and obscure cases can be clarified.

The monophyly of the genus *Sarsia* (Corynidae) was supported by our molecular data with one exception: one *Sarsia* sp. specimen from the North Sea nested within *Stauridiosarsia* species. This may be a result of incorrect identification because of morphological similarity among corynid genera. Since the species identity of this *Sarsia* sp. specimen (KC440084) was not specified, uncertainty of its relationships with other *Sarsia* specimens can be expected. Additionally, two identical 16S sequences from GenBank associated with different species names (*S. tubulosa*: EU876548 and *S. princeps*: EU876549) appeared to be doubtful, and two distinct sequences, EU876548 (*S. tubulosa*, inventory number “MHNGINV35763”) and AJ878720 (*S. tubulosa*, inventory number “Natural History Museum of Geneva INVE 35763”), were unlikely to have been obtained from the same specimen. The COI sequence of *S. princeps* (S30) from the White Sea was 99.9% identical (one substitution) to that of *S. principes* from GenBank (GQ120061). The 16S sequence from the same specimen (S30) was only 98% similar (12 substitutions) to the doubtful EU876549 sequence. Therefore, we propose that the EU876548 and EU876549 sequences are related to neither *S. tubulosa* nor *S. princeps*. The medusa specimen (MG422634) from Canadian waters grouped with *S. lovenii*. We suggest that this specimen was misidentified as *S. princeps* since it was collected during the barcoding project (BioProject: PRJNA472144) and the quality of the presented photo (BOLD:CNNN028-08. COI-5P) is not sufficient for species identification. This finding also may indicate that *S. lovenii* hydroids produce medusae in Canadian waters as well as in the White Sea.

Our phylogenetic analysis supported the validity of six *Sarsia* species: *S. tubulosa*, *S. lovenii*, *S. princeps*, *S. apicula*, *S. striata* and *S. bella*. The status of the other four morphologically valid species, *S. densa*, *S. occulta*, *S. piriforma*, and *S. viridis*, should be verified after molecular sampling. Genetic interspecific distances in *Sarsia* hydrozoans are small in comparison with those in the closely related genus *Coryne* (Ref.^36^; Supplementary Information Table S2), which should be considered during species delimitation.

Most species of the genus *Sarsia* produce free medusae^28^. Only one species of *S. lovenii* produces fixed eumedusoids, which exhibit umbrella and gastral canals but do not have tentacles or ocelli. This species was designated as a member of genus *Sarsia* due to the presence of a long manubrium, the location of gonophores, the occurrence of cnidocyst haplonemes, etc.^28^. Our novel results regarding the ability of *S. lovenii* to produce free-living medusae similar to other *Sarsia* species further support its affiliation with this genus.

The question of whether closely related species can produce different types of gonophores was a key question in hydrozoan systematics at the species or generic levels^4,11^. Hydrozoan taxonomists have long underestimated the frequency of medusa reduction during evolution. Allman (1864) and many followers argued that the degree of gonophore development from a fixed sporosac to a free-living medusa should be used to distinguish different hydrozoan genera^37–41^. Recently, molecular phylogenetic analyses were able to show that closely related species can produce highly divergent types of gonophores^9,11^; thus, the presence/absence of a gonophore type is not an appropriate criterion for classifying genera. Nevertheless, the systematics of some hydrozoan families have not been improved accordingly^42^. Our results suggest the necessity of taxonomical revisions of those hydrozoan taxa.

Another taxonomical extreme was put forth by Louis Agassiz (1862), who proposed the hypothesis of life cycle plasticity (polyphemism of gonophores) in *Coryne mirabilis*^25^. The hypothesis was supported by data from several other hydrozoan scientists^24,25,43,44^. But their observations were discounted due to confusion in the identification of corynid hydrozoans^26,31,45^. Many ideas on this topic were summarized by C. Edwards (1978), who argued the independent status of *S. tubulosa* and *S. lovenii* using experimental observations^32^. C. Edwards concluded “… I have studied many colonies of both [species] in aquaria under controlled and varied temperature conditions. They are evident specifically distinct”. Our data support the independent status of *S. tubulosa* and *S. lovenii*. In experiments of C. Edwards *Sarsia tubulosa* hydroids produced medusae at temperatures within the range 2–20 °C. *Sarsia lovenii* hydroids produced medusoids at temperatures within the range 5.4–10.6 °C. We collected medusae *S. tubulosa* mainly in summer, when the temperature of the surface of the sea reached maximal values of 10–17 °C. Nevertheless in one case colony *S. tubulosa* produced medusa bud (S39) in aquarium under temperatures of 4–6 °C. Hydroids *S. lovenii* with medusoids may be collected in the White Sea when the temperature increases to 5–10 °C. These observations are congruent with C. Edwards experiments on this species^32^. However, in an aquarium we registered the appearance of medusoids (haplogroup 1: specimen S34) after increasing of the temperature from 0–1 °C to 2–5 °C. We suggest that development of medusoids in the White Sea initially starts at temperatures of 2–5 °C but prolongs at higher temperatures. In the White Sea *S. lovenii* hydroids produce medusae mainly after decreasing of the temperature to negative or 0 °C values. Nevertheless, in an aquarium *S. lovenii* colonies began to produce medusae buds in a broader temperature range from 0 to 6 °C (Supplementary Information Fig. S7). For example we observed hydroids from haplogroup 2 (specimen MSU9) produced medusae buds at temperatures of 4–6 °C. These observations indicate that type of gonophore in two haplogroups of *S. lovenii* does not depend on temperature.

We identified two haplogroups of *S. lovenii* with different types of gonophores: hydroids of *haplogroup 1* produce medusoids, and hydroids of *haplogroup 2* produce free medusae. The species identity of two haplogroups was supported by several methods of species delimitation such as phylogenetic approach, ABGD method and partly biological approach. We demonstrated the possibility of two-way interbreeding between medusoid and medusa specimens of *S. lovenii* from the White Sea. The hydroids of two haplogroups inhabit the same locality in the White Sea (Supplement Material Table S1, Fig. S1) and occur sympatrically at least at the polyp stage. Two haplogroups of *S. lovenii* do not pass the test of reciprocal monophyly. We infer that hydrozoans of different haplogroups can undergo crossing in the sea because the discordance of the nuclear and mitochondrial sequences of specimen MSU4 (Table 1) was a result of such an interbreeding event. Finally, ABGD test confirmed that genetic distances between two haplogroup *S. lovenii* correspond to intraspecies distances within other *Sarsia* species such as *S. tubulosa*. Nevertheless, interbreeding between two haplogroups of *S. lovenii* is likely a rare event in the White Sea because the only one specimen among 33 examined was the offspring of such crossing.

Crossing between specimens from different *S. lovenii* haplogroups resulted in formation of hybrids with intermediate phenotype. The outsprings in our experiments produced normal medusae buds with tentacles and ocelli (Supplementary Information Fig. S2). But gonophores formed gonad being retained on the colony. We have not found gonophores with such morphology in the White Sea, but similar gonophores are known from previous works. Louis Agassiz studied corynid hydrozoans in Massachusetts Bay and figured an “almost perfect male medusa”, which as he thought was permanently attached to colony and withering after discharging sperm^25^. The similar colonies from the Gulf of Maine were later figured by N.J. Berrill^43^. Finally such hydrozoans from Scotland were described by C. Edwards as a new species, *S. occulta*^32^. According to C. Edwards *S. occulta* hydroids retain gonophores until the gonads are partly developed but liberate them as medusae for further growth and maturation. But in the one case *S. occulta* gonophores formed ripe gonads before detachment from the colony^32^. In our experiments we failed in feeding of hybrid medusae by nauplii of *Artemia*. Medusae did not grow but they became ripe before detachment. After detachment medusae slowly swam near the bottom of the bowl similarly to behaviour described for *S. occulta*^32^. Our results emphasize that species status of *S. occulta* still should be verified with extensive molecular sampling. Another explanation of existence of those gonophore types in nature is intra- or interspecific hybridization events. Since free-living medusae of *S. lovenii* were found only in Resolute Bay in Canada (a specimen with accession GB number MG422634) or in the White Sea, and according to our experimental data we assume hydroids of *S. lovenii* normally produce free-living medusae only in the cold Arctic waters. We have not any information on appearance of *S. lovenii* medusae in boreal areas, i.e. Massachusetts Bay or in the waters of Scotland. Findings of gonophores with intermediate phenotype in these regions may be a result of hybridization between different corynid species. For instance, A. Brinckmann-Voss indicated the possibility of hybridization between two *Sarsia* species, *S. tubulosa* and *S. apicula*^46^.

Summarize all data we can interpret the existence of two haplogroups with different gonophore’s types within species *S. lovenii* as a case of nascent speciation. Existence of two sympatric haplogroups *S. lovenii* infers some isolation mechanism which may limit crossing between haplogroups. We can propose three possible mechanisms: differences in daily spawning timing, differences in the spawning season, and spatial distance between spawning specimens. R. L. Miller and A. Brinckmann-Voss suggested that the time difference in spawning might be a potential isolation mechanism in sympatric *Sarsia* species^46,47^. However, the daily changes in illumination in the White Sea in June are insignificant, and we did not observe any daily periodicity in the spawning of the studied specimens. For example, medusoid females spawned clutches of eggs in both day and night time. Differences in the spawning season may limit free interbreeding in some years (Fig. 6). Appearing of medusae in plankton depends on environment conditions in winter or in the early spring, while the formation of medusoids affected by environmental conditions in the late spring. Timing of gonophores development and spawning may be uncorrelated in medusae and medusoids and it results in crossing or isolation events in different years. Another explanation comes from spatial differences in habitat of spawning specimens: free-living medusae spawned broadly in water column, while medusoids produce gametes locally near the bottom. In this case the possibility of crossing between medusae and medusoids depends on longevity of gametes and chances of their meeting in plankton. Further discussion concerning the crossing rate between the two haplogroups in the sea may be continued after detailed estimation of haplotype frequencies.

## Conclusion

In the present paper, we have shown that *Sarsia lovenii* presents polymorphism of its life cycle, producing both medusae and medusoids. This polymorphism is congruent with the observed genetic divergence and population structure, which may be interpreted as being a result of reproductive isolation and nascent speciation. Nevertheless, successful interbreeding between two haplogroups in our experiments indicates that reproductive barriers are not absolute. Further dedicated studies are needed to understand the processes underlying this intraspecific life cycle polymorphism. The taxonomic value of medusa reduction is ambiguous and is applied at different taxonomic levels when in different hydrozoan families, such as Companulariidae, Bougainvilliidae, Corynidae, etc.^4,9,41,48^. Reduction of medusae at the species level has been shown by molecular phylogenetic methods for many hydrozoans; however, this character is still used for classifying genera. Variation of life cycle strategies within *S. lovenii* demonstrates that closer attention should be paid to similar cases within different hydrozoan lineages. The recent revival of life cycle evolution studies^49,50^ and progress in molecular methods contribute to further insight into hydrozoan evolution, but the understanding of the mechanism of medusa reduction is still far from comprehensive. The hydrozoan *S. lovenii* represents a potential model system for examining the mechanism of the gain or loss of life-history stages.

## Materials and Methods

### Sample collection

Medusae and hydroids of *Sarsia* spp. (39 specimens) were collected near the White Sea Biological Station (WSBS) in Kandalaksha Bay (66°34′N, 33°08′E) (Supplementary Information Fig. S1; Table S1). Medusae were sampled near the water surface close to the pier of WSBS and from the water column with a plankton net in adjacent localities. Corynid hydroids were collected in the intertidal zone (Eremeevskie rapids) near WSBS from various substrata, including stones and kelp (*Fucus* sp.*, Ascophyllum* sp.). Medusa buds were found on the specimens collected in March-April, and medusoids were found on the specimens collected in May-June. Additional material (13 specimens) was collected at WSBS from a flow-in aquarium system with free input of water from the sea or from a closed aquarium system. Throughout the year, the temperature in the intertidal zone (Eremeevskie rapids) was measured every hour by a DST-CTD data logger (Star-Oddi Ltd.). All material was preserved in 96% EtOH.

### Maintaining of hydroids in laboratory

Colonies *Sarsia* sp. were transported to department of Invertebrate Zoology (Moscow State University) and maintained all-the-year in aquaria with artificial sea water (salt Red Sea Pro, salinity 25–27‰). Polyps were fed by *Artemia* nauplii. To encourage the production of gonophores, temperature in the aquaria was lowered to 0–2 °C or 4–6 °C.

### Morphological analysis

Specimens were examined under an Olympus SZ51 stereomicroscope. We photographed all collected specimens alive, including colonies, individual polyps, medusa buds, medusoids, and adult medusae. Photographs were taken with a Canon D550 camera supplied with a Canon macro 100 mm or Canon MP-E macro lens. The tentacle bases of most medusae were photographed from both front and side views. Photographs of all specimens are presented in supplements (Supplementary Information Fig. S2). Morphological characteristics were drawn from photographs in accordance with the molecular identification of species.

### Molecular analysis

*Taxon sampling*. Forty-eight specimens from the White Sea were used in the phylogenetic analysis. Additionally, 35 sequences of *Sarsia* species available in GenBank were included (Supplementary Information Table S3). Outgroups were chosen mainly according to Nawrocki *et al*.^35^ and considering the availability of both COI and 16S sequences in GenBank. Sequence of *Stauridiosarsia producta* from the White Sea was used as outgroup for the phylogenetic analysis of ITS rRNA fragments.

#### DNA extraction and sequencing

DNA was extracted using the Promega Wizard SV Genomic DNA Purification kit (Promega Corporation, Madison, USA) or Diatom DNA Prep 100 kit (Isogen Lab, Moscow, Russia) according to the manufacturer’s protocol. Extracted DNA was used as a template for the amplification of partial cytochrome *c* oxidase subunit I (COI), *16S* rRNA (16S), and internal transcribed spacer region (*ITS1* and *ITS2*) of the rRNA gene cluster (ITS) using the primers and program indicated in Supplementary Information Table S4. Polymerase chain reaction amplifications were carried out in a 20-µL reaction volume, which included 4 µL of 5x Screen Mix (Evrogen Joint Stock Co., Moscow, Russia), 0.5 µL of each primer (10 µL stock), 1 µL of genomic DNA and 14 µL of sterile water. The Promega PCR Purification Kit protocol (Promega) was used to purify the amplification products. Amplification of products proceeded in both directions. Each sequencing reaction mixture contained 1 μL of BigDye (Applied Biosystems, PerkinElmer Corporation, Foster City, CA), 1 μL of 1 μM primer, and 1 μL of DNA template; sequencing reactions were run for 40 cycles of 96 °C (15 s), 50 °C (30 s), and 60 °C (4 min). Sequences were subjected to ethanol precipitation to remove unincorporated primers and dyes. The products were resuspended in 12 μL of formamide and subjected to electrophoresis in an ABI Prism 3500 Genetic Analyzer (Applied Biosystems) at the N.K. Koltsov Institute of Developmental Biology (Moscow, Russia) or the N.A. Pertsov White Sea Biological Station MSU (Primorsky, Russia).

All new sequences were deposited in GenBank under accession numbers (MN240175-MN240289) (Supplement Information Table S1). DNA of all collected specimens is available upon request to the corresponding author.

#### Phylogenetic analysis

Sequences were assembled and checked for improper base calling with CodonCode Aligner software (www.codoncode.com/aligner). Sequences were aligned using the MUSCLE^51^ algorithm in MEGA 6 software^52^. The final alignments yielded fragments of 661, 689, and 764 bp for the 16S, COI and ITS loci, respectively. A test of substitution saturation was carried out with Damble^53,54^.

Individual marker analyses and a concatenated analysis were performed. Each gene was analysed independently to check for incongruence between trees. JModelTest 2^55^ was used to estimate the best substitution model for each partition based on the Bayesian information criterion (BIC). The HKY + I + G model^56^ was found to be optimal for the 16S dataset; the GTR + G + I model^57^ was found to be optimal for the COI dataset; and the HKY + G model^56^ was found to be optimal for the ITS dataset.

Maximum likelihood analyses (ML) were performed using Garli 2.0^58^ according to the optimal models for each gene. A search for the best maximum likelihood tree was conducted along with bootstrap analysis (1000 bootstrap pseudoreplications). Bootstrap values were placed on the best tree found with SumTrees 3.3.1 from DendroPy Phylogenetic Computing Library Version 3.12.0^59^.

Bayesian phylogenetic trees were built in PhyloBayes 3.3^60^. The analysis was initiated with random starting trees and 7 million generations. Two MCMC chains were run in parallel, and the analyses were stopped when the maximum discrepancy of bipartitions between chains was below 0.01. Final phylogenetic tree images were rendered in FigTree 1.4.0.

After visual inspection of the absence of supported incongruence between the resulting trees, a concatenated COI + 16S + ITS dataset was prepared. The concatenated analysis was limited to specimens from the White Sea for which at least COI sequences were obtained and those specimens from GenBank that had both COI and 16S gene sequences available. The concatenated COI + 16S + ITS alignment, which was constructed from 60 specimens, yielded a sequence alignment of 2088 bp. Maximum likelihood analyses (ML) of the concatenated dataset were performed using Garli2.0 with 1000 bootstrap pseudoreplications. A Bayesian phylogenetic tree for the concatenated dataset was built in PhyloBayes 3.3 with random starting trees and 11 million generations.

A haplotype network was constructed using the TCS network inference method^61^ within PopART software (http://popart.otago.ac.nz/index.shtml). According to a constraint of the method, we used a reduced COI dataset without undefined states of nucleotides.

#### Species delimitation analysis

Minimum and maximum uncorrected *p*-distances were calculated with MEGA 6 software. We also applied the Automatic Barcode Gap Discovery (ABGD) method to detect species-level clusters. ABGD is a distance-based method designed to detect the so-called ‘barcode gap’ in the distribution of pairwise distances calculated in a COI alignment^62^. The web-based ABGD program (available at http://wwwabi.snv.jussieu.fr/public/abgd/) was employed with the default settings (P = 0.001–0.1 and X = 1.5) to generate a preliminary partition of sequences using the COI alignment and excluding outgroups.

### Crossing experiments

Medusae and colonies with medusoids were collected in the middle of June 2018 near WSBS. Experiments were carried out in glass bowls (200 ml) at 10–12 °C using filtered (0.2 µm) sea water. Individuals of each morphotype (medusa or medusoid) were paired for spawning with the opposite sex of the same or different morphotype (Supplementary Information Table S5). Experiments were carried out with five replications. The specimens spawned in ambient conditions in the laboratory without specific stimulation. The offspring of two successful experiments (experiment 1: male_medusa_S37 × female_medusoid_S38; experiment 2: male_medusoid_S35 × female_medusa_S36) were reared in the laboratory and used for molecular analyses (specimens S43-S51).

## Supplementary information

Figure S1-S7

Table S1-S5

## Data Availability

All data generated or analysed during this study are included in this published article (and its Supplementary Information Files) or are available from the corresponding authors upon reasonable request.
