# Supplementary material for: A case of nascent speciation: unique polymorphism of gonophores within hydrozoan Sarsia lovenii
Source: Sci Rep. 2019 Oct 29;9:15567. doi: 10.1038/s41598-019-52026-7 (PMC6820802; doi:10.1038/s41598-019-52026-7)

## Supplementary Information File

**A case of nascent speciation: unique polymorphism of gonophores within hydrozoan *Sarsia lovenii***

Andrey A. Prudkovsky*, Irina A. Ekimova, Tatiana V. Neretina

***Correspondence:** Andrey Prudkovsky, aprudkovsky@wsbs-msu.ru

Figure S1. Sampling localities in the White Sea

Figure S2. Photographs of specimens used for phylogenetic analyses. Full description of specimens presents in Table S1

Figure S3. Bayesian and Maximum Likelihood phylogenetic hypothesis, based on mitochondrial (COI, 16S) and nuclear (ITS) datasets

Figure S4. Detection of barcode gap in the distribution of pairwise distances (ABGD) calculated in the COI alignment for *Sarsia* spp

Figure S5. Results of crossing experiments

Figure S6. Variation of shape of medusa apical appendage

Figure S7. Photographs of gonophores which appeared on colonies *Sarsia sp.* in laboratory

**Figure S1.** Sampling localities in the White Sea. The base geographic layer (USGS/NASA's Landsat 8 satellite data) was downloaded from the Landsat 8 satellite database (http://libra.developmentseed.org, accessed 29 May 2016). Landsat imagery courtesy of NASA Goddard Space Flight Center and U.S. Geological Survey. The base geographical layer of inset was generated using OpenStreetMap (© OpenStreetMap contributors, under the Open Database License. https://www.openstreetmap.org/copyright/en).

Abbreviations: WSBS, White Sea Biological Station; 1, pear of WSBS; 2, Eremeevskie rapids; 3, saline lake at Green Cape; 4, Kislo-sladkoe lake

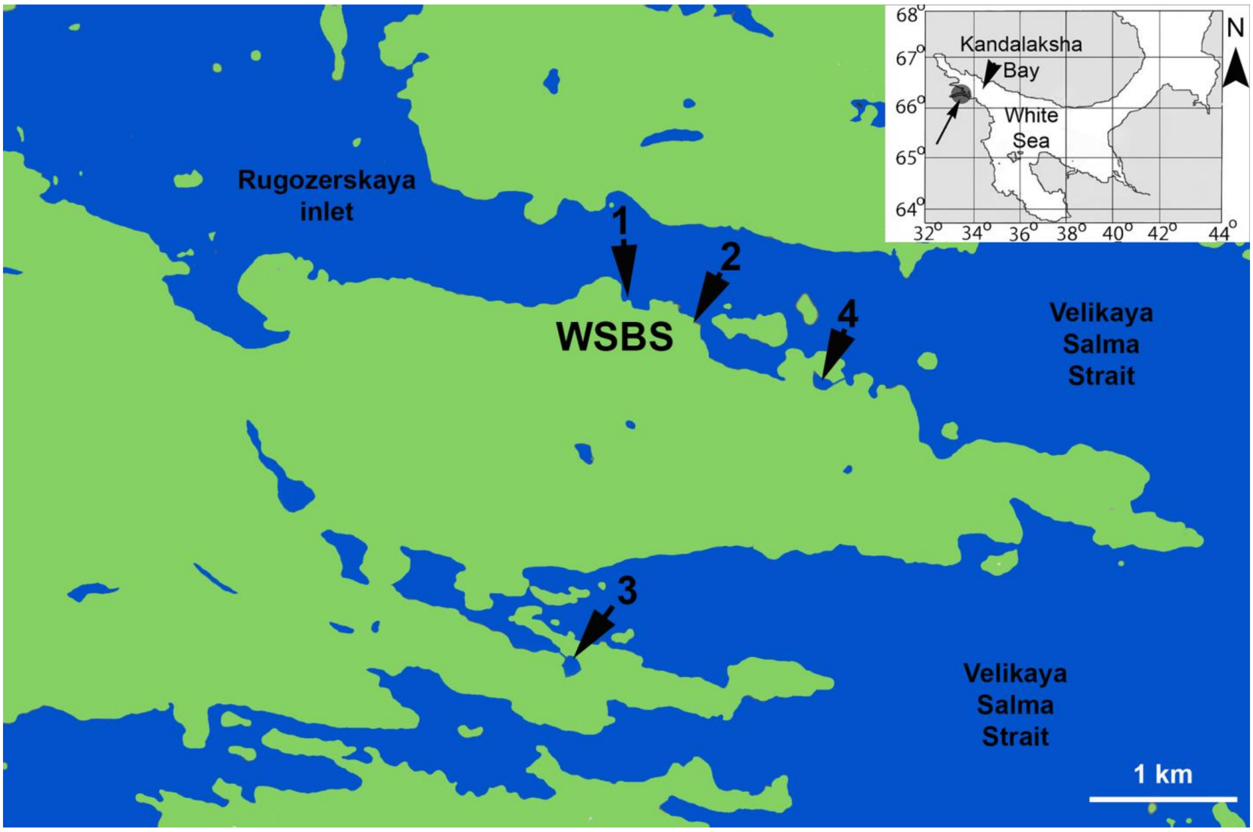

**Figure S2.** Photographs of specimens used for phylogenetic analyses. Full description of specimens present in Table S1. Photographs of specimens EV7-EV15 were kindly provided by Marfa Everett.

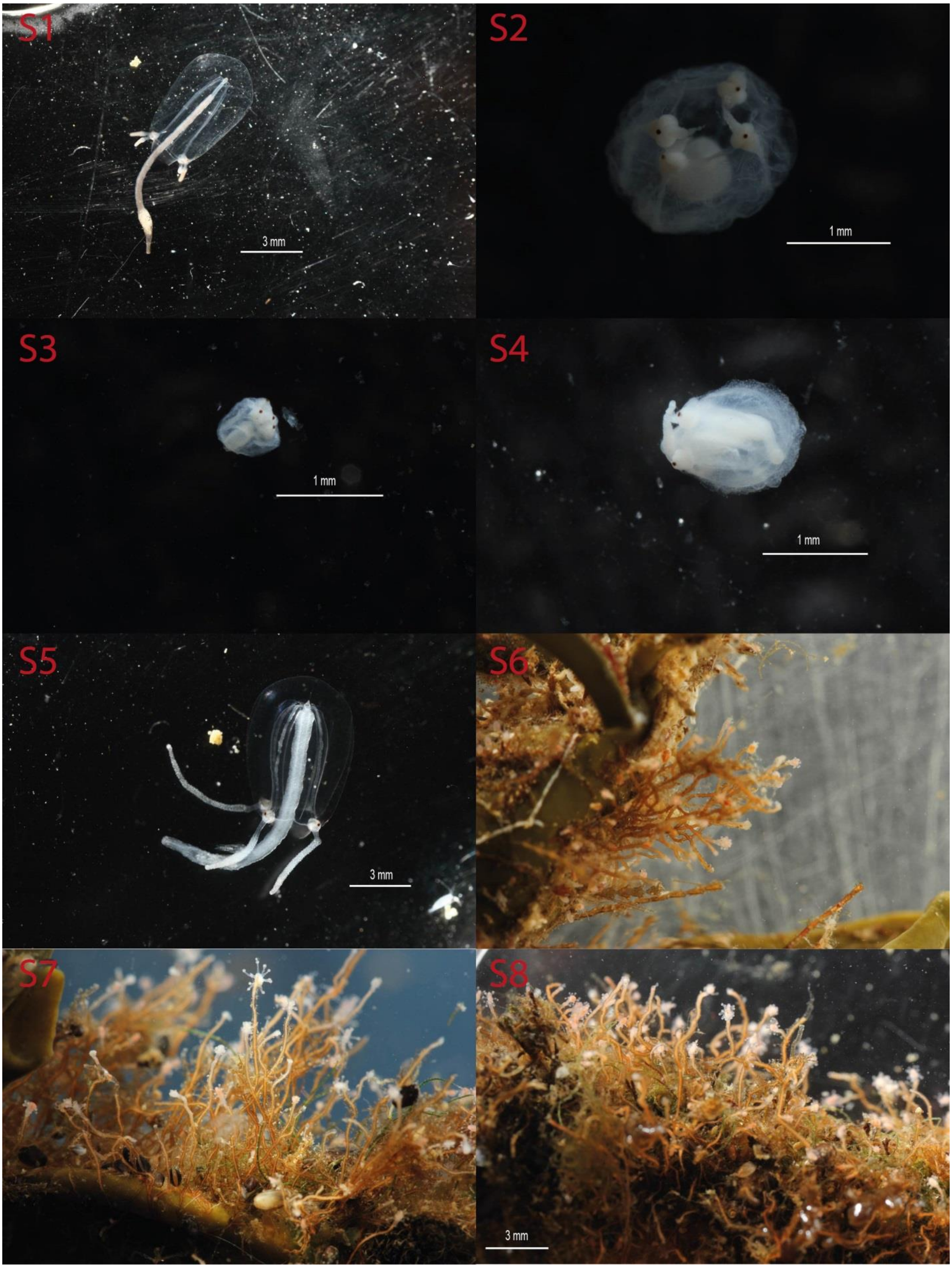

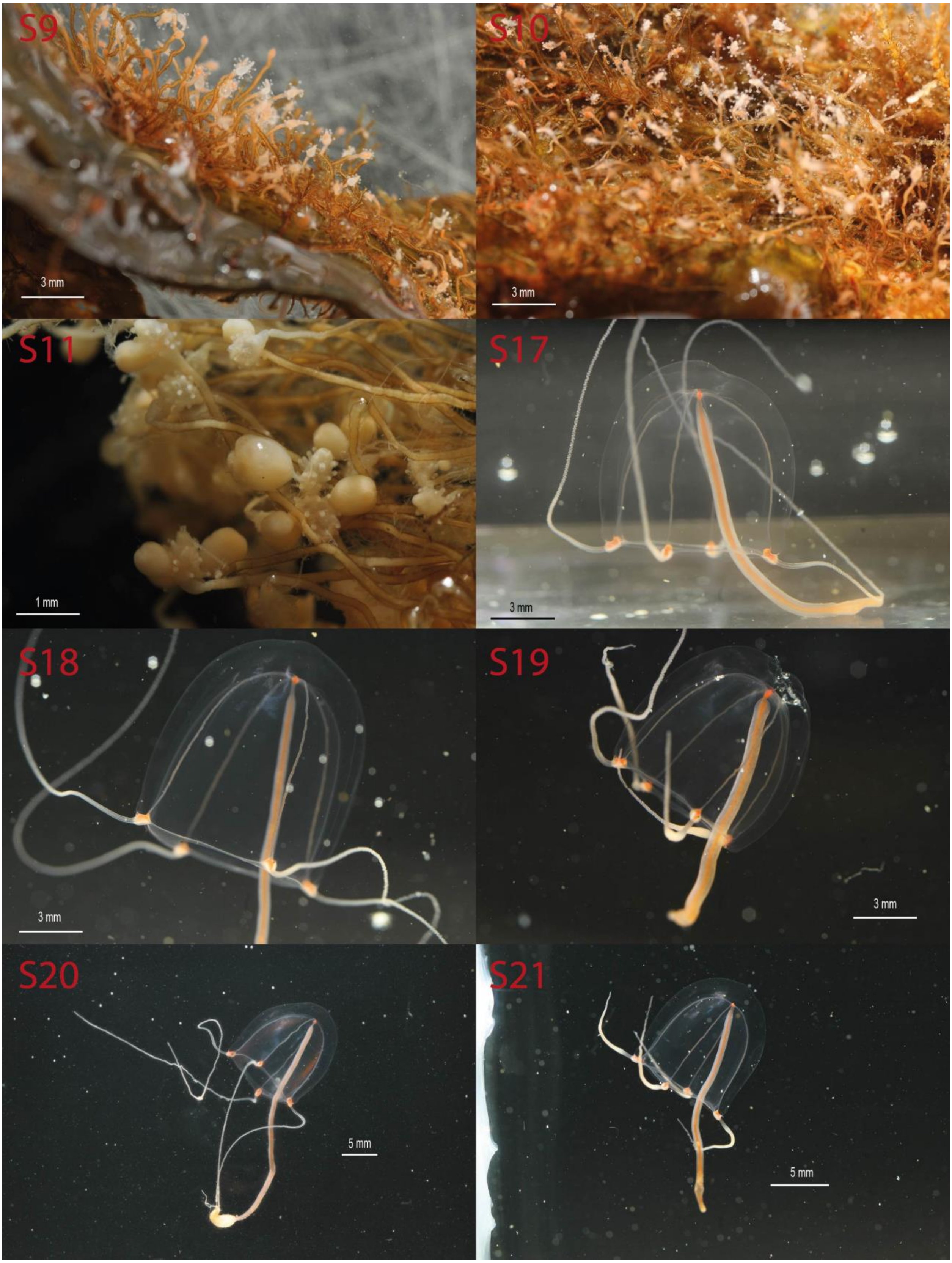
S9
3 mm
S10
3 mm
S11
1 mm
S17
3 mm
S18
3 mm
S19
3 mm
S20
5 mm
S21
5 mm

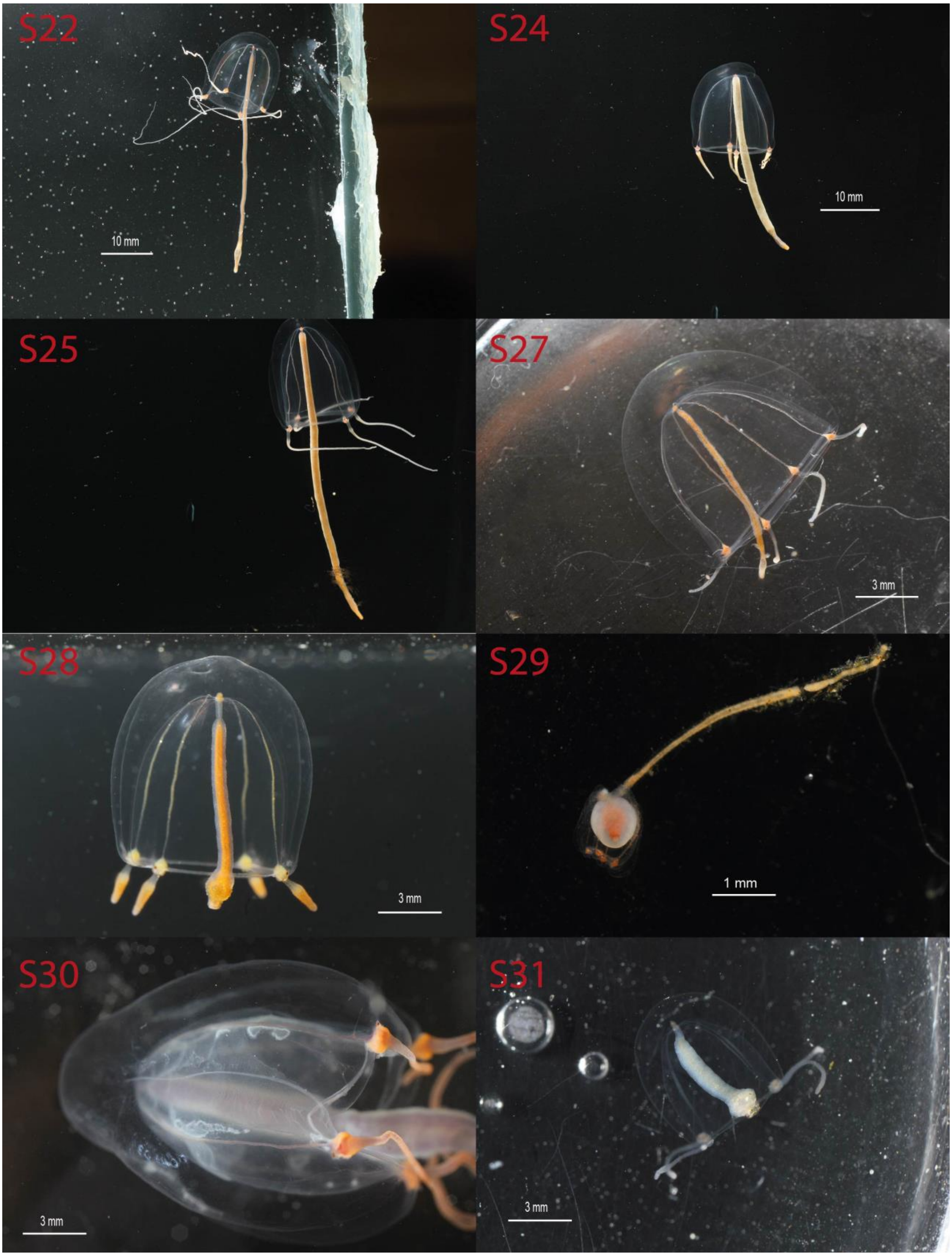
S22
10 mm
S24
10 mm
S25
S27
3 mm
S28
3 mm
S29
1 mm
S30
3 mm
S31
3 mm

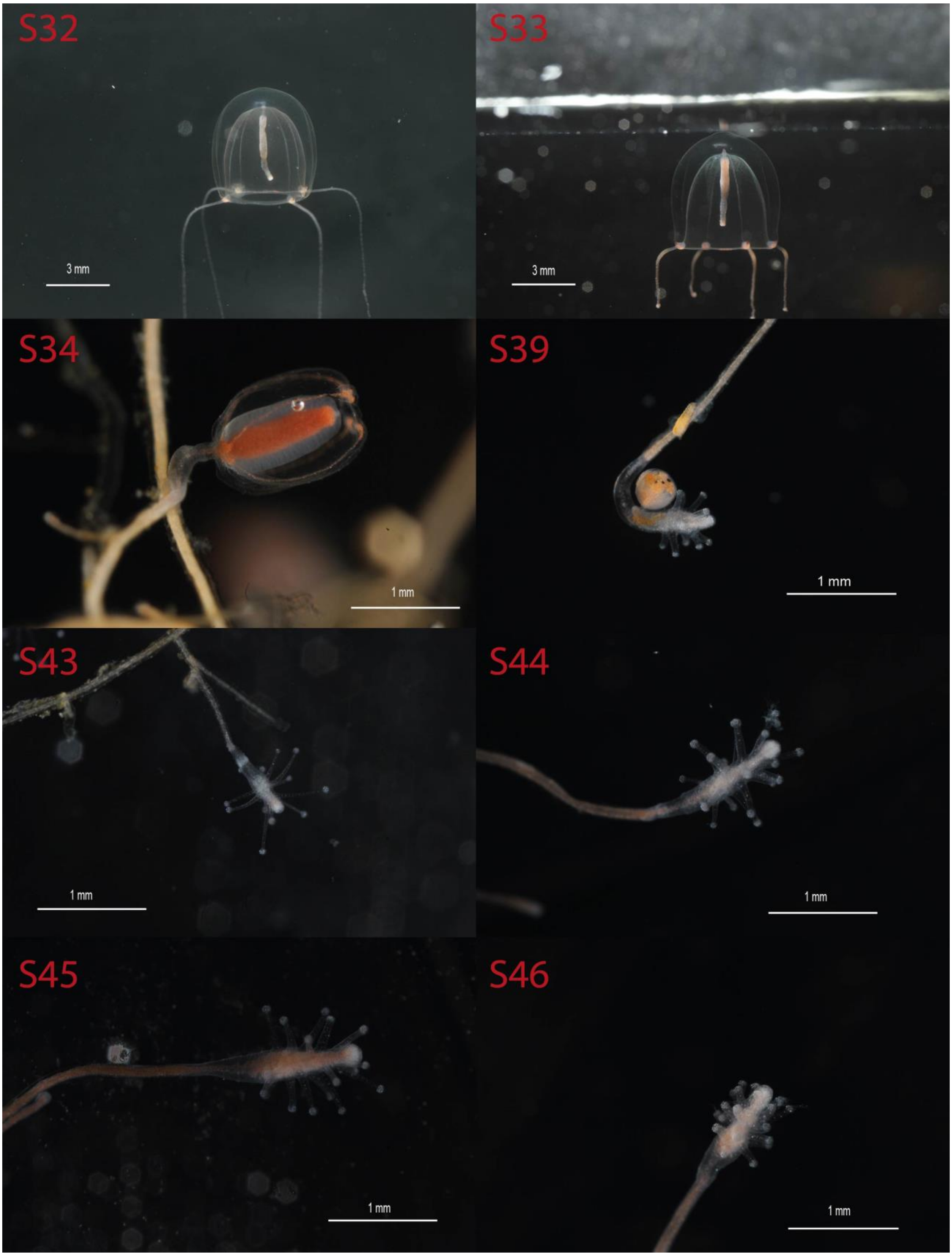
S32
3 mm
S33
3 mm
S34
1 mm
S39
1 mm
S43
1 mm
S44
1 mm
S45
1 mm
S46
1 mm

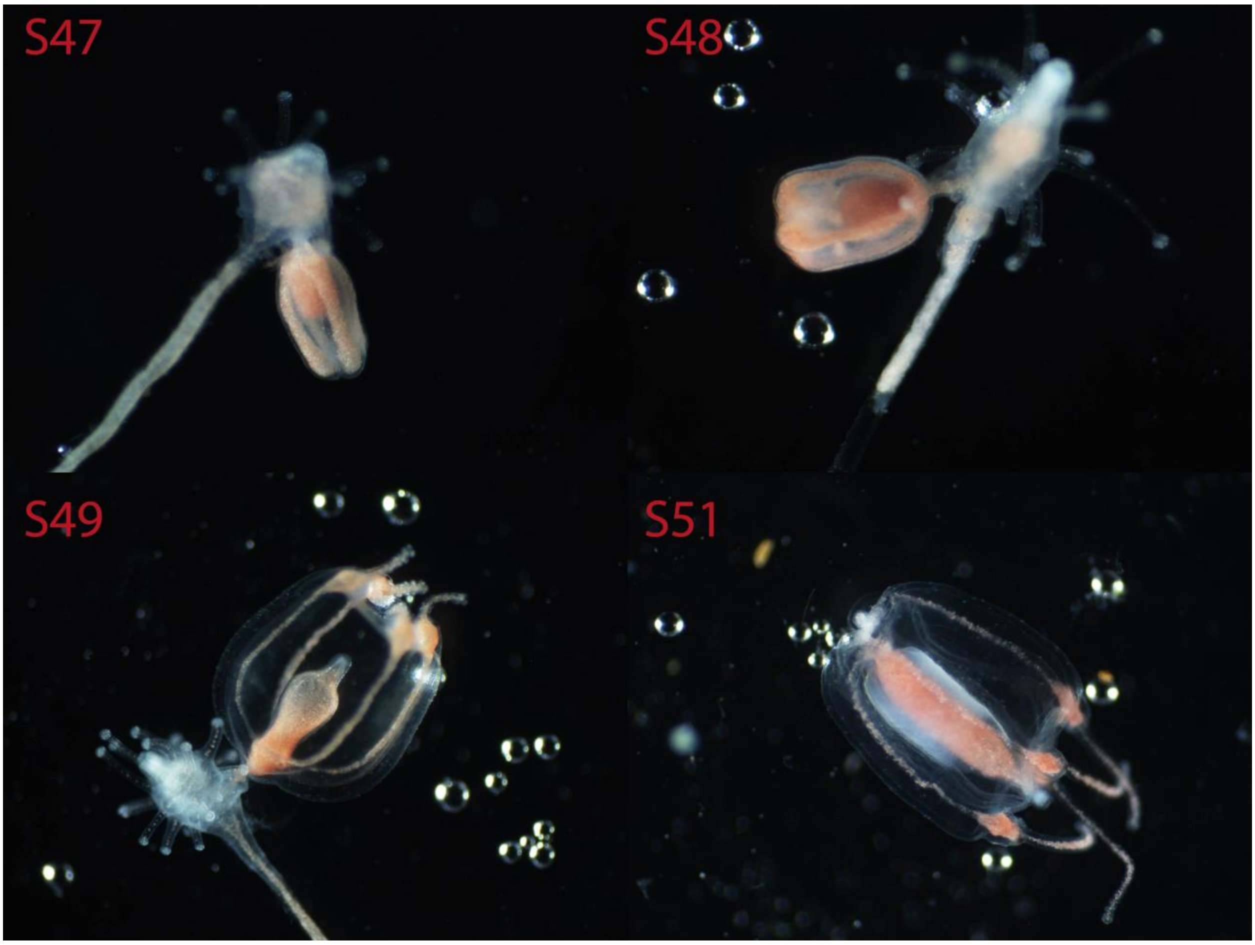
S47
S48
S49
S51

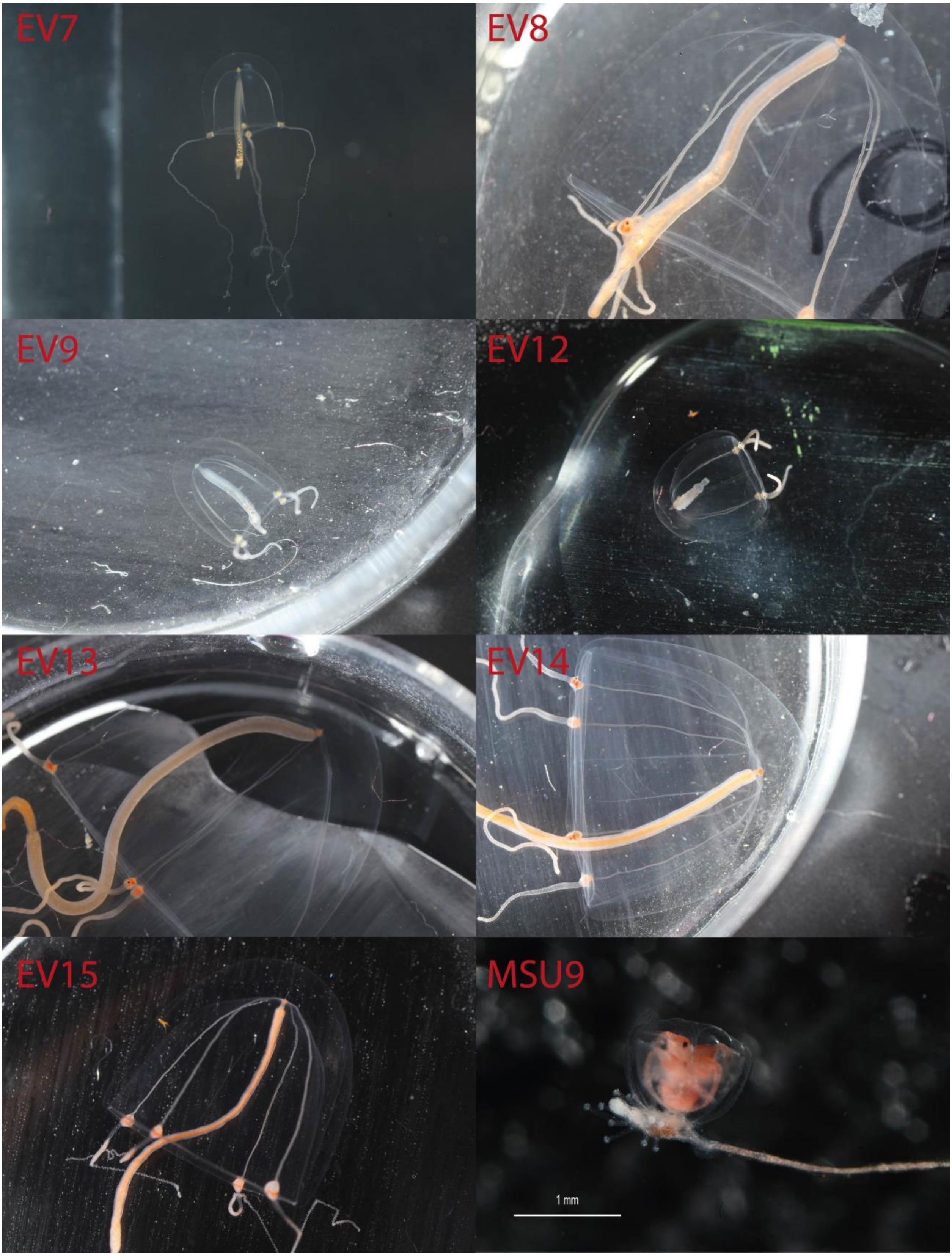
EV7
EV8
EV9
EV12
EV13
EV14
EV15
MSU9
1 mm

**Figure S3.** Bayesian and Maximum Likelihood phylogenetic hypothesis, based on mitochondrial (COI, 16S) and nuclear (ITS) datasets.

Figure S3a. Bayesian phylogenetic hypothesis, based on mitochondrial COI dataset. Numbers on branches represents posterior probabilities from Bayesian Inference.

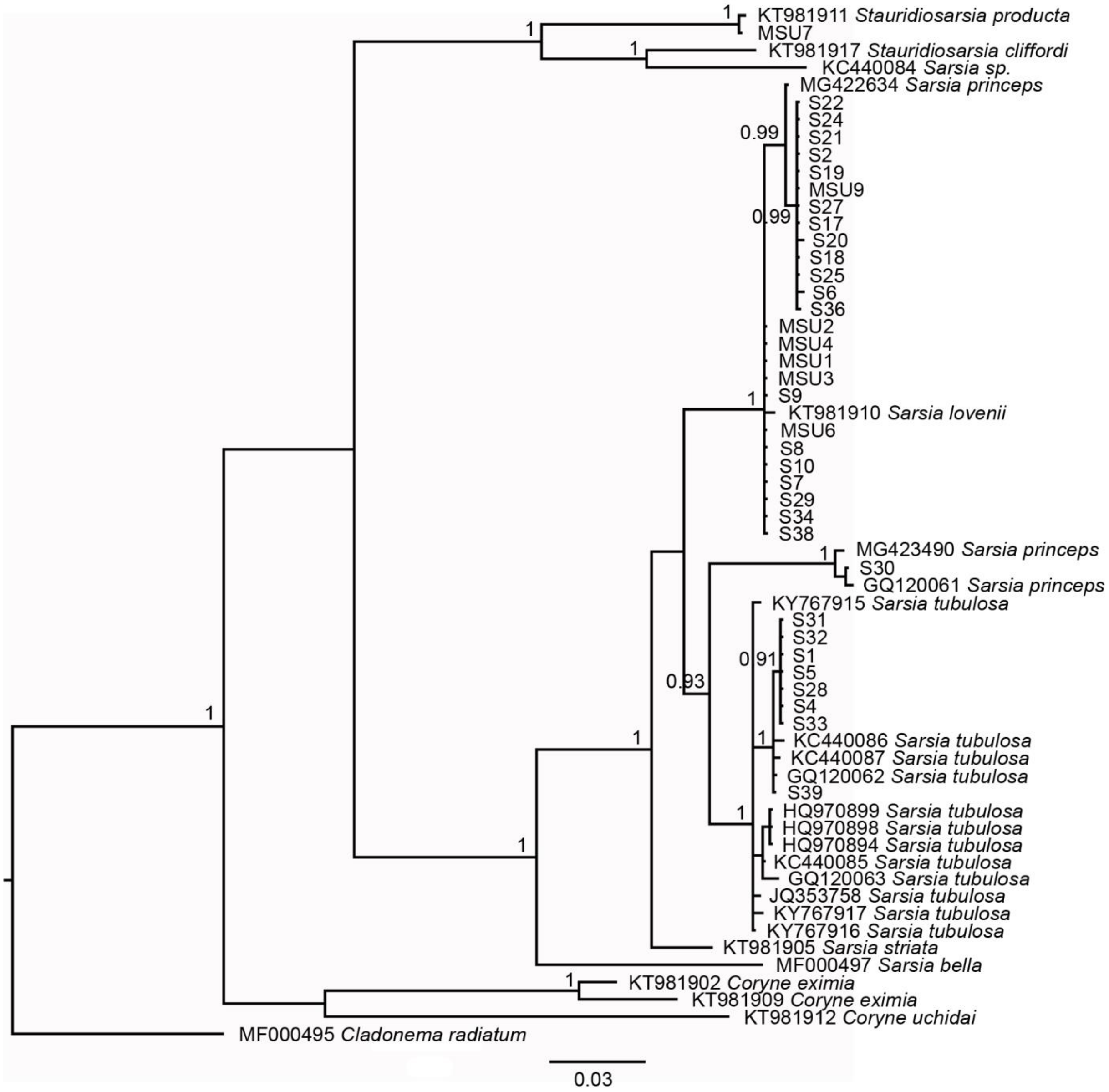

Figure S3b. Maximum Likelihood phylogenetic hypothesis, based on mitochondrial COI dataset. Numbers on branches represents boostrap values from Maximum Likelihood (1000 pseudoreplicates).

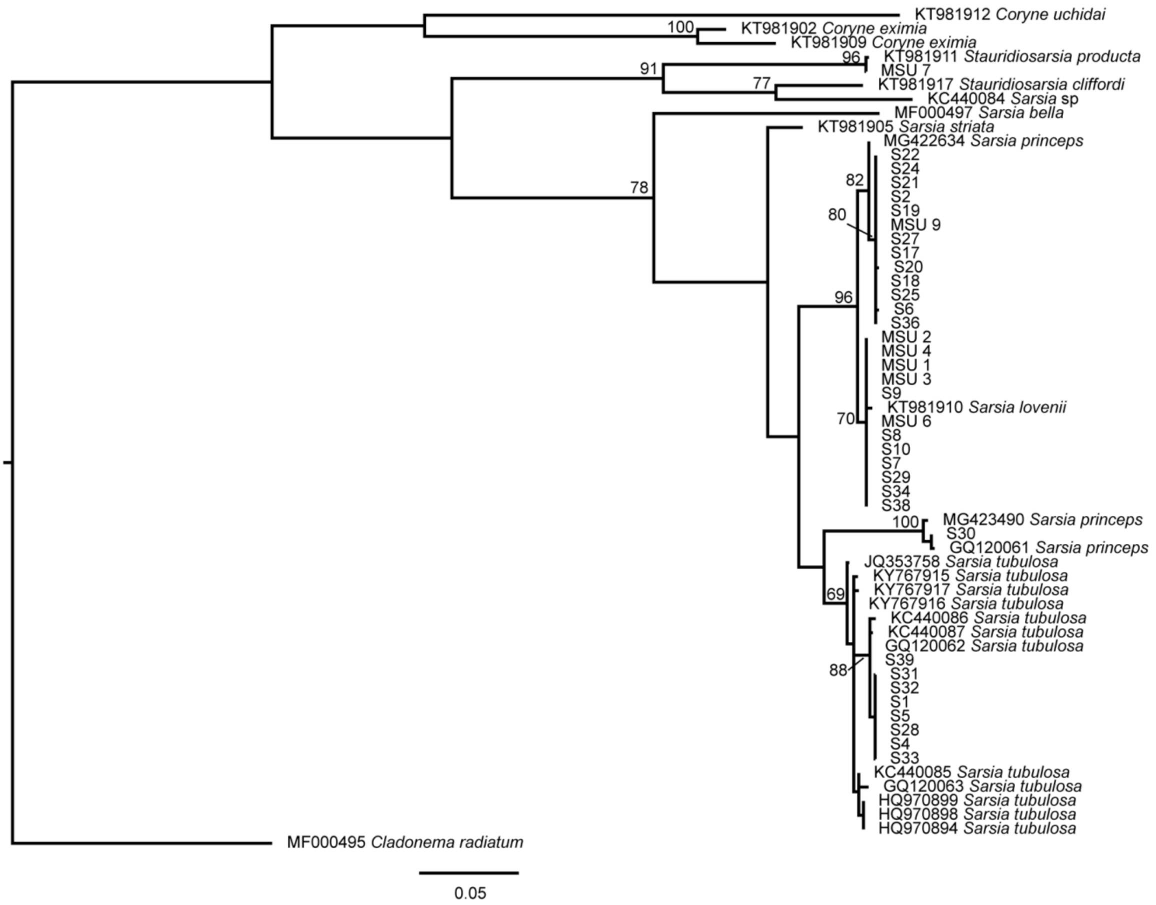

Figure S3c. Bayesian phylogenetic hypothesis, based on mitochondrial 16S dataset. Numbers on branches represents posterior probabilities from Bayesian Inference.

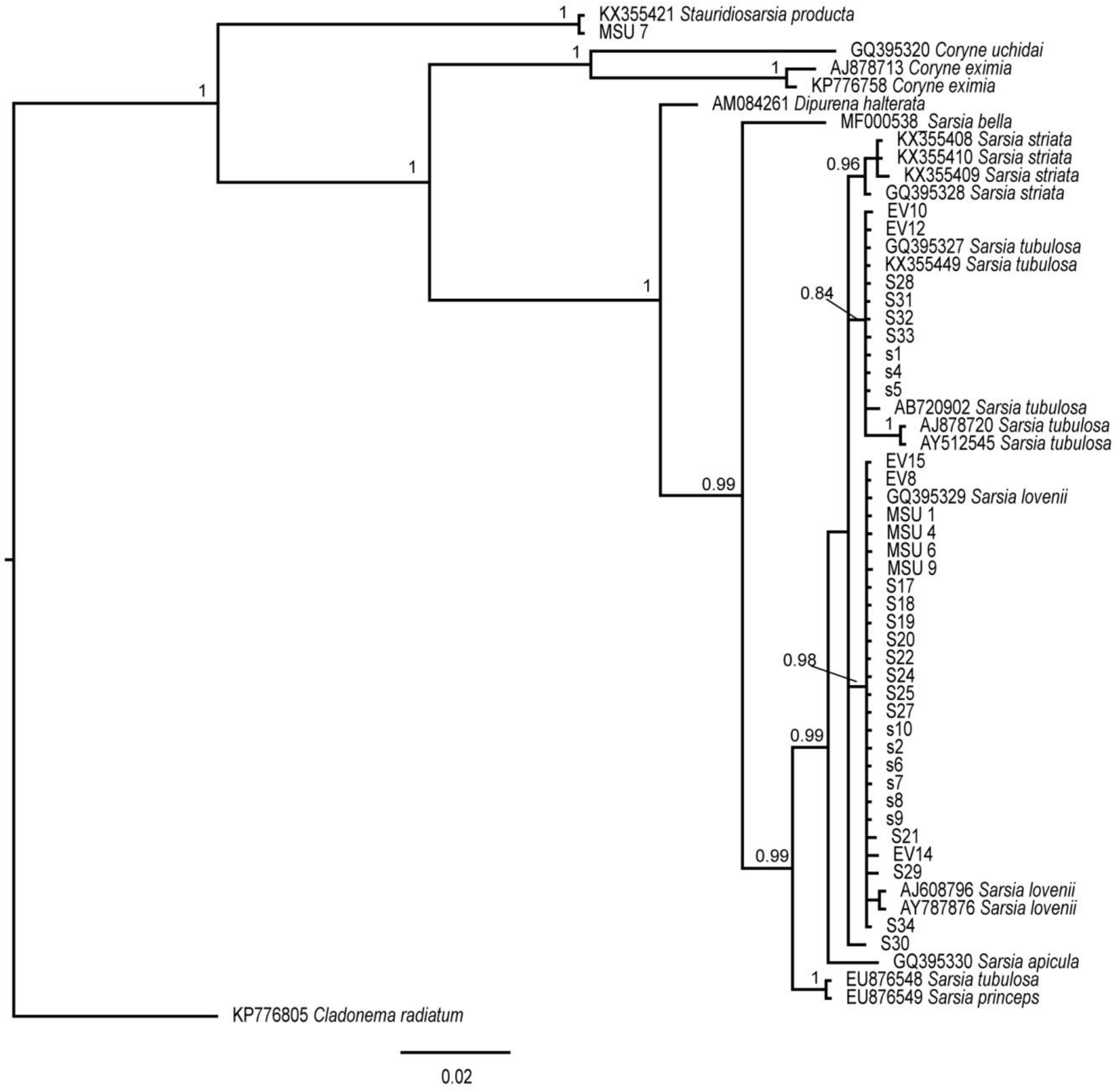

Figure S3d. Maximum Likelihood phylogenetic hypothesis, based on mitochondrial 16S dataset. Numbers on branches represents boostrap values from Maximum Likelihood (1000 pseudoreplicates).

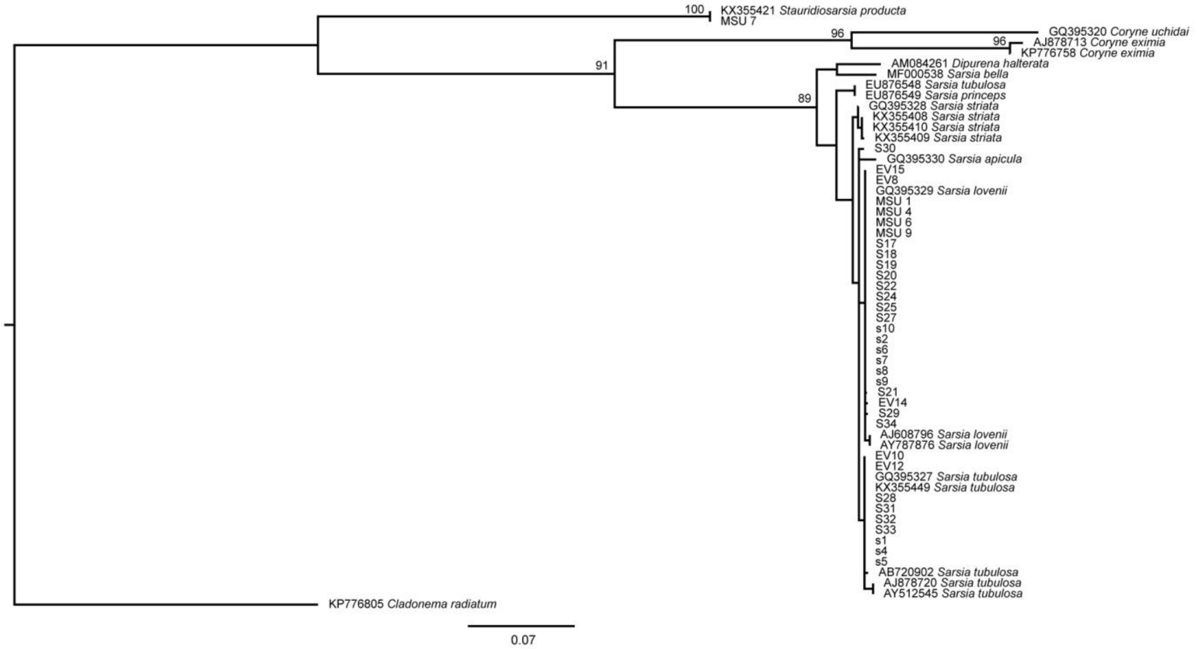

Figure S3e. Bayesian phylogenetic hypothesis, based on nuclear ITS dataset. Numbers on branches represents posterior probabilities from Bayesian Inference.

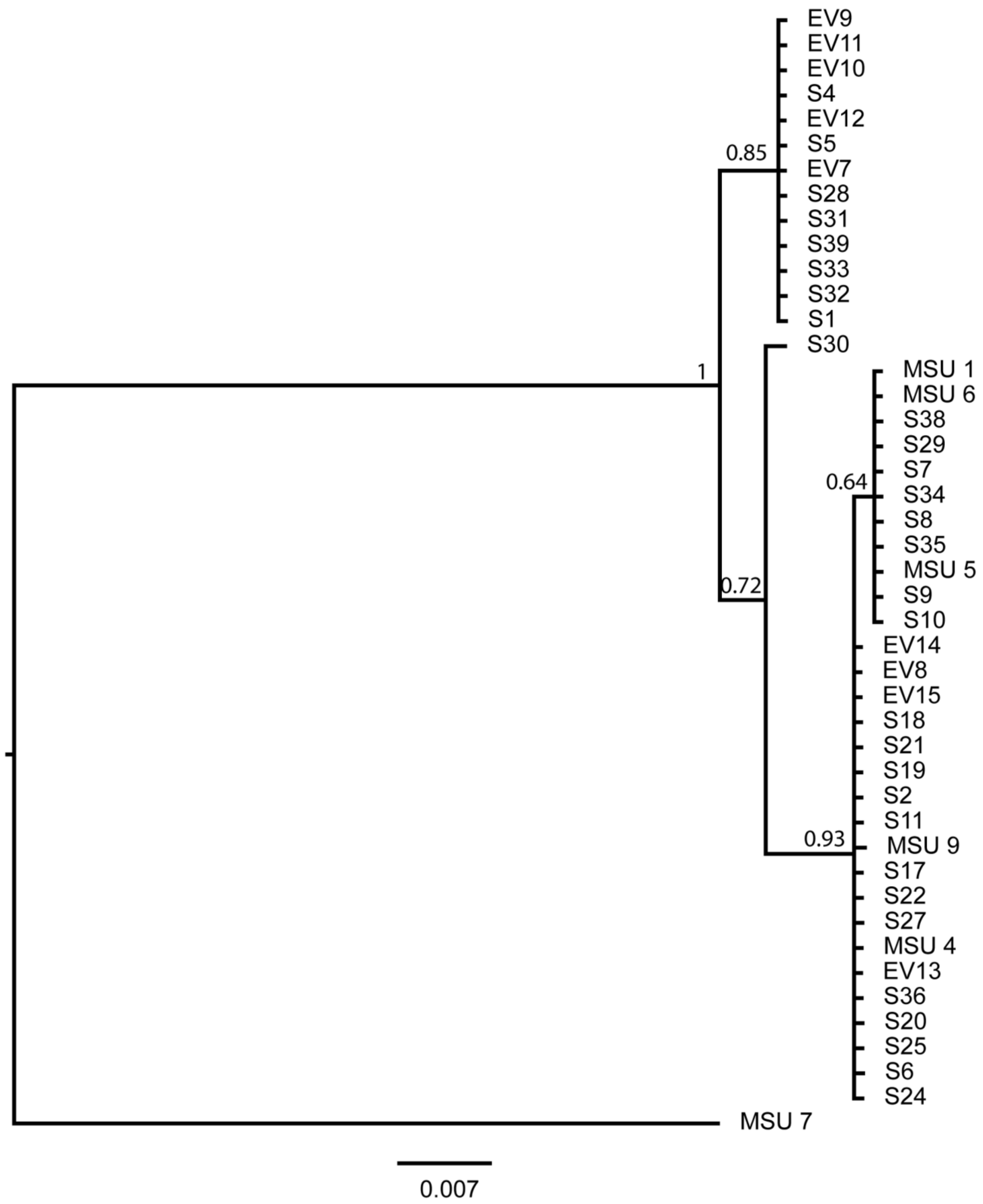

Figure S3f. Maximum Likelihood phylogenetic hypothesis, based on nuclear ITS dataset. Numbers on branches represents boostrap values from Maximum Likelihood (1000 pseudoreplicates).

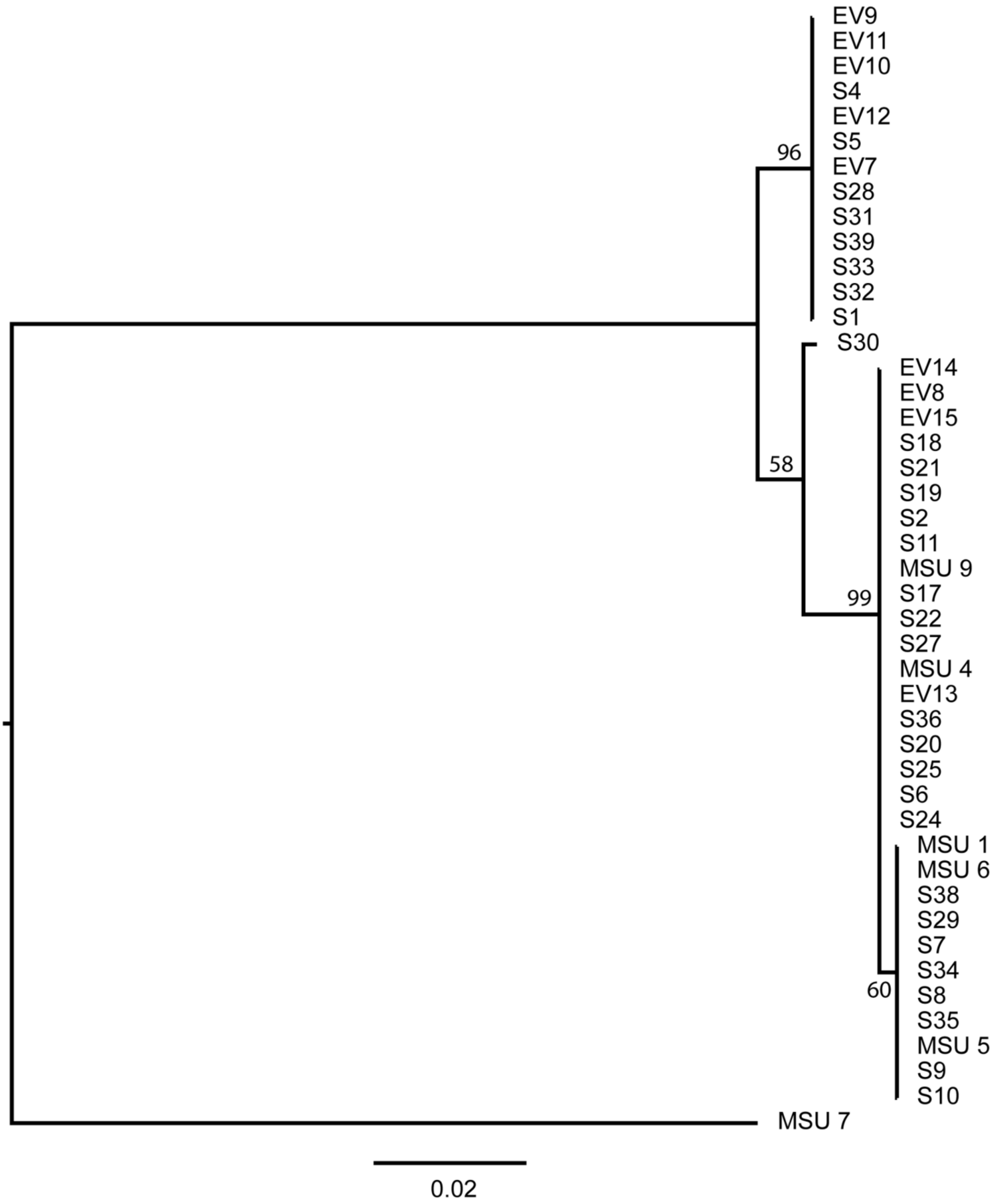

**Figure S4.** ABGD-web results for COI alignment of *Sarsia* spp. using JC69 Jukes-Cantor model (A), K80 Kimura model (B), and “simple distances” model (C) as measure of distance. Y-axis represents a number of recovered species-level groups, depending on different Prior intraspecific divergence P (X-axis). Yellow squares indicate groups recovered from the initial partition, red square – from the recursive partition. Diagram on the right represents the histogram of calculated distances. The barcode gap, separating intra and interspecific distance values, is indicated with the red arrow. Default settings: P=0.001-0.1 and X=1.5.

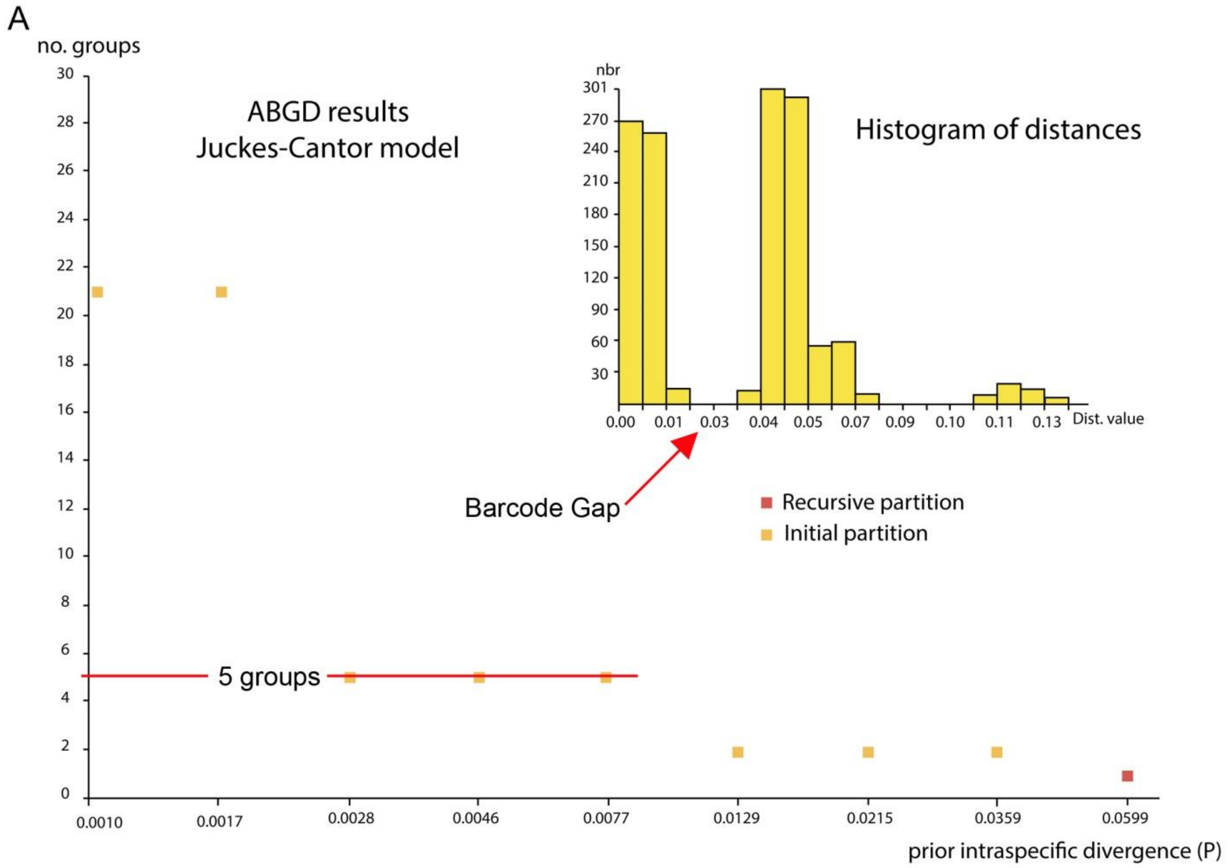

B

C

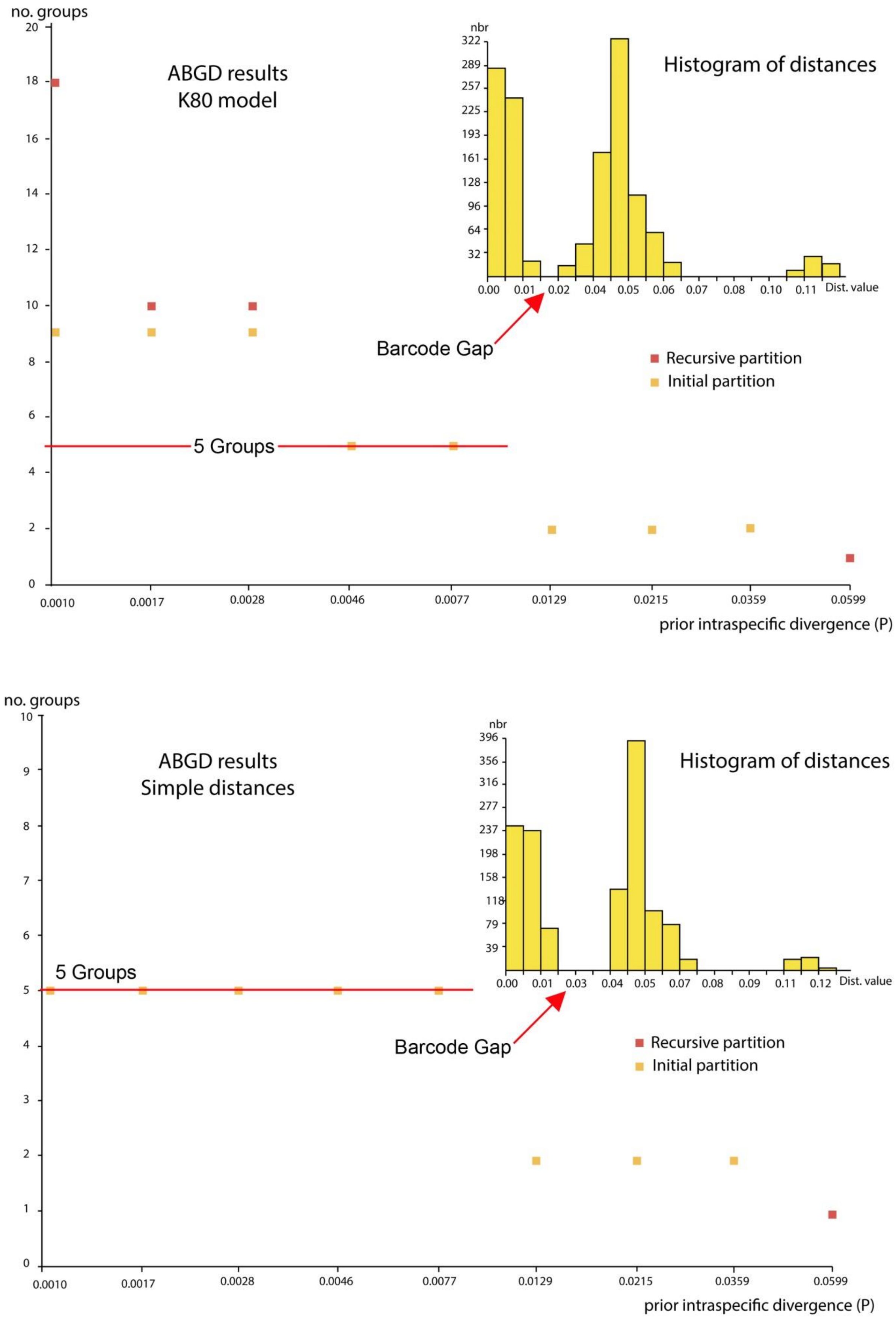

no. groups
ABGD results
K80 model
Histogram of distances
nbr
Dist. value
Barcode Gap
Recursive partition
Initial partition
5 Groups
prior intraspecific divergence (P)
0.0010
0.0017
0.0028
0.0046
0.0077
0.0129
0.0215
0.0359
0.0599
no. groups
ABGD results
Simple distances
Histogram of distances
nbr
Dist. value
5 Groups
Barcode Gap
Recursive partition
Initial partition
prior intraspecific divergence (P)

**Figure S5.** Results of crossing experiments:

- A-D. Stages of cleavage. - E-F. Planula larvae.

- B-E. Male medusa x female medusoid. - A, F. Female medusa x male medusoid.

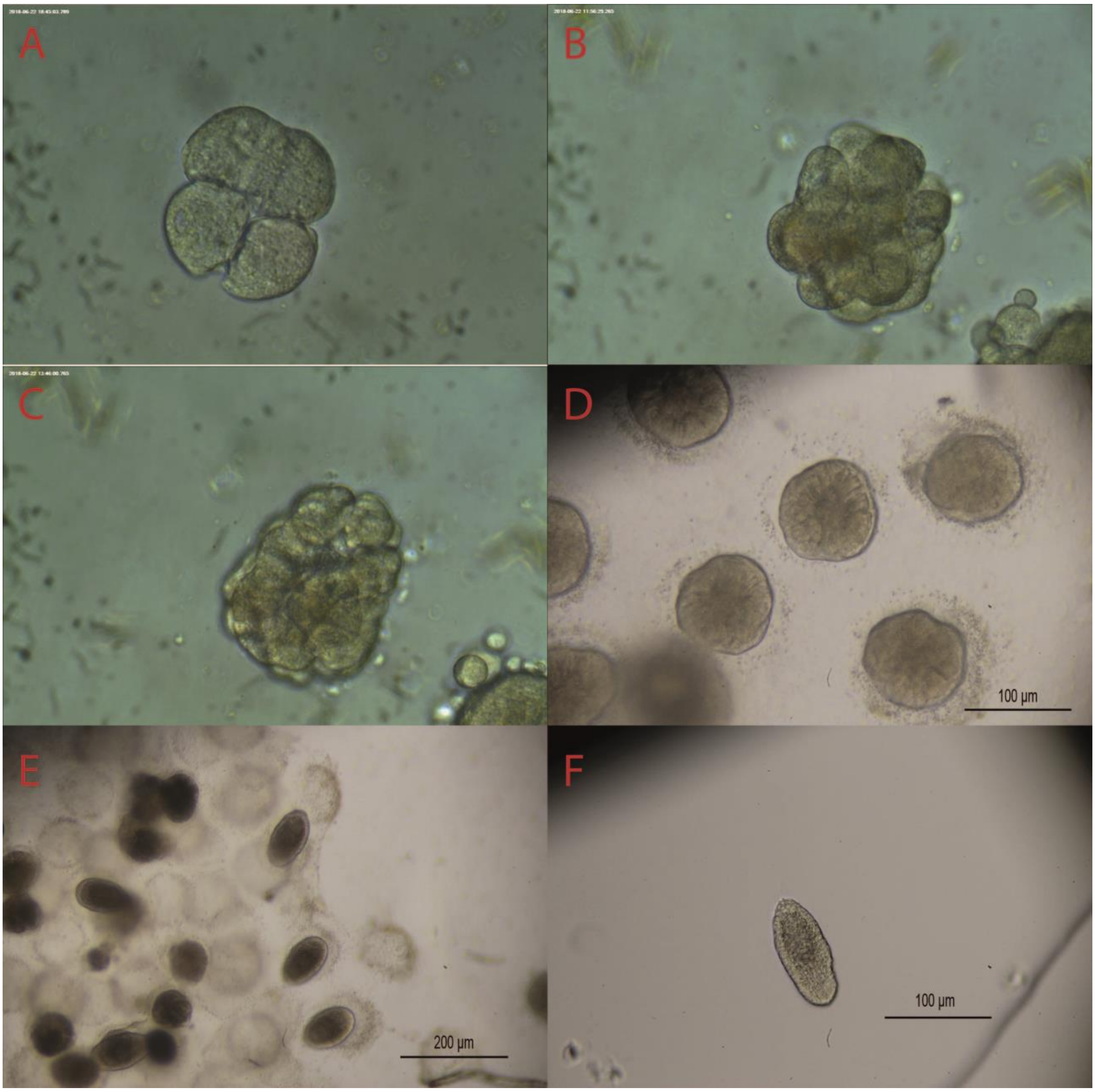

**Figure S6.** Variation of shape of medusa apical appendage: **a** *Sarsia lovenii*; **b** *Sarsia tubulosa*

Abbreviations: ac – apical canal; ach – apical chamber; ex.s. – exumbreal surface; g – gonad; mg – mesoglea; pm – proximal part of manubrium; rad.c. – radial canal; sf – subumbrellar fold

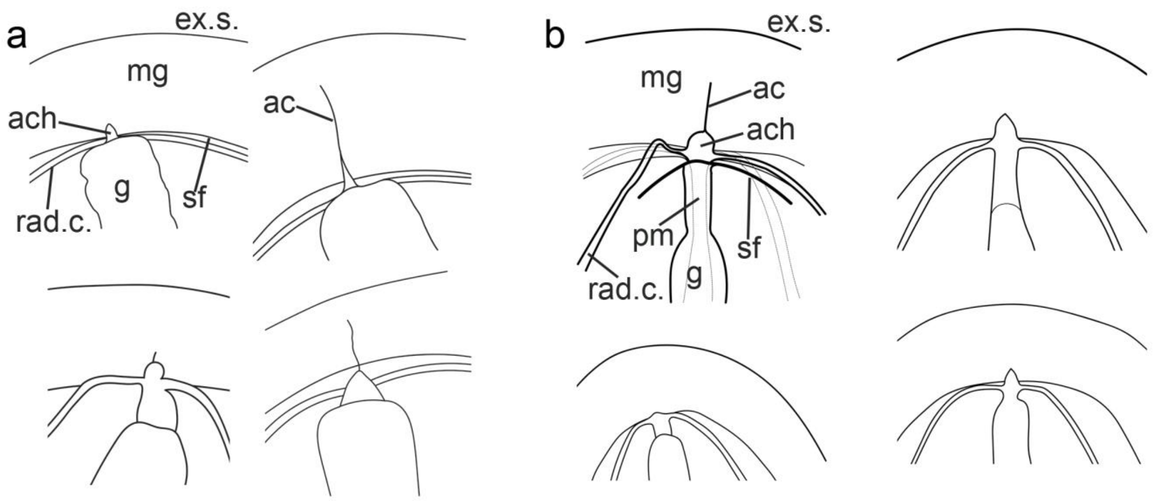

**Figure S7.** Photographs of gonophores which appeared on colonies *Sarsia* sp. in laboratory: **a** Newborn medusa, date 28.01.2015, temperature 4-6 °C. **b** Polyp with medusa bud, date 11.03.2017, temperature 0-2 °C. **c** Polyp with medusa bud, date 18.03.2017, temperature 4-6 °C. **d** Newborn medusa, date 26.04.2017, temperature 0-2 °C. **e** Polyp with medusa bud, date 18.01.2018, temperature 0-2 °C. **f** Newborn medusa, date 30.04.2019, temperature 0-2 °C. **g** Specimen MSU9, date 26.03.2015, temperature 4-6 °C. **h** Specimen S34, date 22.05.2018, temperature 2-5 °C.

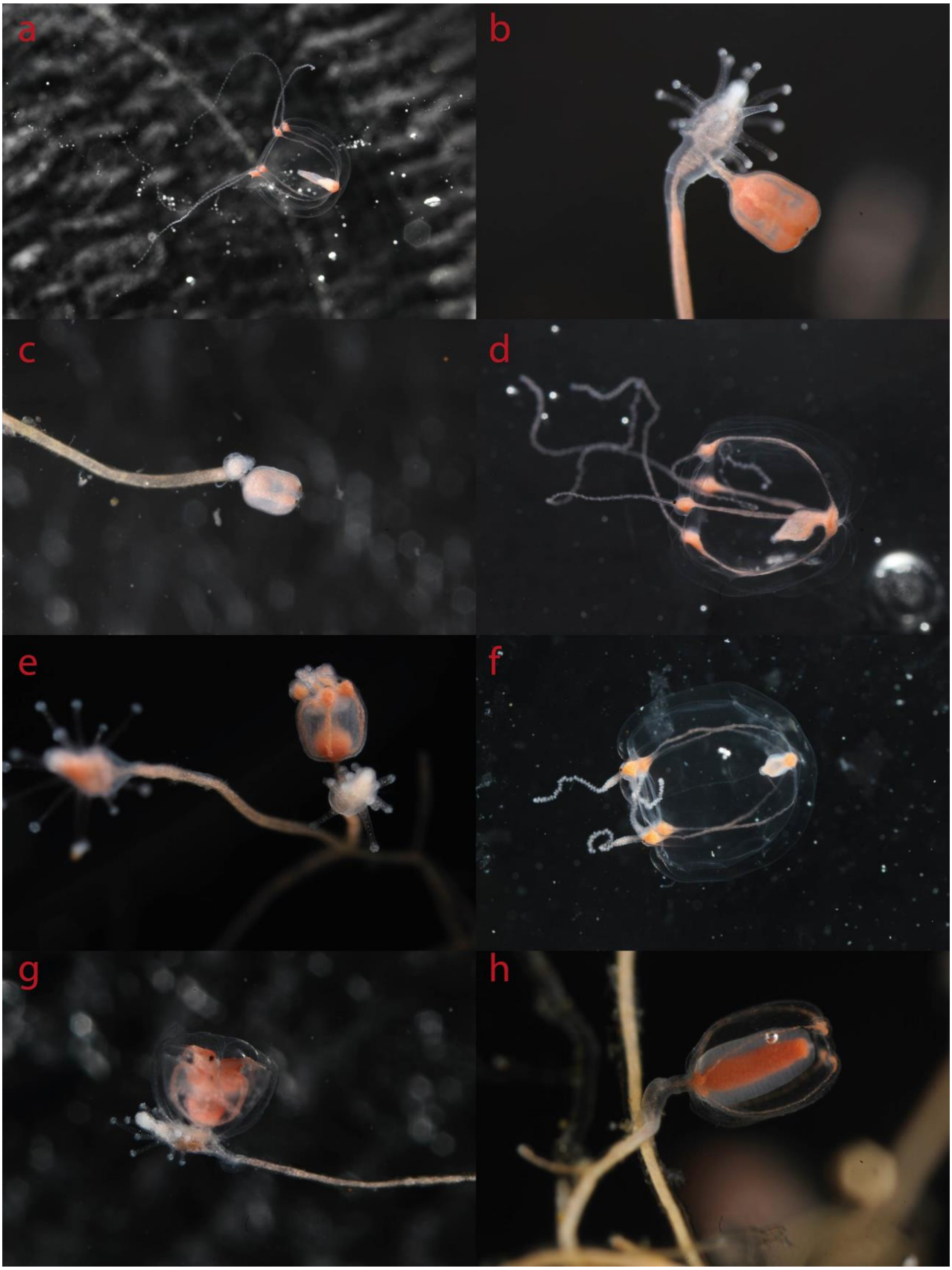

Supplement: Supplementary file 1 — Figure S1-S7 [file 41598_2019_52026_MOESM1_ESM.pdf]
