# Supplementary material for: A case of nascent speciation: unique polymorphism of gonophores within hydrozoan Sarsia lovenii
Source: Sci Rep. 2019 Oct 29;9:15567. doi: 10.1038/s41598-019-52026-7 (PMC6820802; doi:10.1038/s41598-019-52026-7)
Supplement: Supplementary file 2 — Table S1-S5 [file 41598_2019_52026_MOESM2_ESM.pdf]

Supplementary Information File

A case of nascent speciation: unique polymorphism of gonophores within hydrozoan *Sarsia lovenii*

Andrey A. Prudkovsky*, Irina A. Ekimova, Tatiana V. Neretina

***Correspondence:** Andrey Prudkovsky, aprudkovsky@wsbs-msu.ru

Table S1. List of corynid specimens from the White Sea used for phylogenetic analyses

Table S2. Range of intraspecific and interspecific *p*-distances for 16S and COI gene fragments for *Coryne* species (after Schuchert, 2005) and *Sarsia* species (*S. tubulosa* and *S. lovenii*)

Table S3. List of specimens from GenBank used for phylogenetic analyses

Table S4. Primers and programs used in amplification reactions

Table S5. Crossing scheme of interbreeding experiments for *Sarsia lovenii*

Table S1. List of corynid specimens from the White Sea used for phylogenetic analyses.

Isolate/Species	Stage	Locality	Data	Temperature °C [1]	GenBank accession number		
					16S	COI	ITS
MSU1/S.l.	p	1	26.03.15	-	MN240222	MN240179	MN240249
MSU2/ S.l.	p	1	26.03.15	-	-	MN240176	-
MSU3/ S.l.	p	1	26.03.15	-	-	MN240184	-
MSU4/ S.l.	p	1	26.03.15	-	MN240223	MN240177	MN240282
MSU5/ S.l.	p	1	26.03.15	-	-	-	MN240278
MSU6/ S.l.	p	1	26.03.15	-	MN240224	MN240195	MN240252
MSU7/S.pro.	p	1	26.03.15	-	MN240210	MN240175	MN240245
MSU9/ S.l.	pb	1	26.03.15	4-6	MN240225	MN240190	MN240271
S1/ S.t.	m	2	29.07.16	14-17	MN240217	MN240186	MN240283
S2/ S.l.	m	2	25.04.16	1	MN240235	MN240188	MN240267
S4/ S.t.	m	2	10.08.16	14-16	MN240218	MN240198	MN240254
S5/ S.t.	m	5	26.07.16	10-17	MN240219	MN240187	MN240256
S6/ S.l.	p	3	6.12.16	-1-0	MN240236	MN240203	MN240288
S7/ S.l.	p	3	6.12.16	-1-0	MN240237	MN240204	MN240268
S8/ S.l.	p	3	6.12.16	-1-0	MN240238	MN240199	MN240273
S9/ S.l.	p	3	6.12.16	-1-0	MN240239	MN240191	MN240280
S10/ S.l.	p	3	6.12.16	-1-0	MN240234	MN240202	MN240281
S11/ S.l.	pb	3	20.04.16	0.2-1	-	-	MN240270
S17/ S.l.	m	2	5.06.17	3-5	MN240226	MN240194	MN240275
S18/ S.l.	m	2	5.06.17	3-5	MN240227	MN240197	MN240260
S19/ S.l.	m	2	5.06.17	3-5	MN240228	MN240189	MN240266
S20/ S.l.	m	2	5.06.17	3-5	MN240229	MN240196	MN240286
S21/ S.l.	m	2	5.06.17	3-5	MN240240	MN240183	MN240261
S22/ S.l.	m	2	5.06.17	3-5	MN240230	MN240181	MN240276
S24/ S.l.	m	2	25.06.17	8-9	MN240231	MN240182	MN240289
S25/ S.l.	m	2	25.06.17	8-9	MN240232	MN240201	MN240287
S27/ S.l.	m	4	30.06.17	-	MN240233	MN240193	MN240277
S28/ S.t.	m	4	2.07.17	-	MN240213	MN240192	MN240263
S29/ S.l.	pm	3	30.06.17	9-11	MN240242	MN240205	MN240262
S30/S.pri.	m	2	6.07.17	10-12	MN240243	MN240185	MN240246
S31/ S.t.	m	4	11.07.17	-	MN240214	MN240178	MN240264

S32/ S.t.	m	4	12.07.17	-	MN240215	MN240180	MN240279
S33/ S.t.	m	4	12.07.17	-	MN240216	MN240200	MN240269
S34/ S.l.	pm	1	22.05.18	2-5	MN240244	MN240206	MN240272
S35[2]/ S.l. male	pm	3	18.06.18	5-9	-	-	MN240274
S36[2]/ S.l. female	m	2	18.06.18	5-9	-	MN240207	MN240285
S37[2]/ S.l. male	m	2	18.06.18	5-9	-	-	-
S38[2]/ S.l. female	Pm	3	18.06.18	5-9	-	MN240208	MN240259
S39/ S.t.	pb	1	7.09.18	4-6	-	MN240209	MN240265
S43[2] (S36 x S35)	p	1	23.10.18	-	-	COI[3]	ITS[3]
S44-S46[2] (S38 x S37)	p	1	23.10.18	-	-	COI[3]	ITS[3]
S49[2] (S36 x S35)	pb	1	8.07.19	0-2	-	COI[3]	ITS[3]
S51[2] (S38 x S37)	pb	1	8.07.19	0-2	-	COI[3]	ITS[3]
EV7/ S.t.	m	4	16.06.17	16	-	-	MN240258
EV8/ S.l.	m	4	16.06.17	16	MN240221	-	MN240251
EV9/ S.t.	m	4	16.06.17	16	-	-	MN240247
EV10/ S.t.	m	4	16.06.17	16	MN240211	-	MN240253
EV11/ S.t.	m	4	16.06.17	16	-	-	MN240248
EV12/S.t.	m	4	16.06.17	16	MN240212	-	MN240255
EV13/ S.l.	m	2	16.06.17	6-9	-	-	MN240284
EV14/ S.l.	m	2	16.06.17	6-9	MN240241	-	MN240250
EV15/ S.l.	m	4	16.06.17	16	MN240220	-	MN240257

Abbreviations. **Species:** S.l. – *Sarsia lovenii,* S.t. – *Sarsia tubulosa,* S.pri. – *Sarsia princeps*, S.pro. – *Stauridiosarsia producta*. **Stage:** p-polyp, pb – polyp with medusa bud, m-medusa, pm – polyp with medusoid. **Locality:** 1 - Aquarium, WSBS; 2 - Pier WSBS; 3 - Eremeevskie rapids; 4 - Saline lake at the Green Cape; 5 - Kislo-sladkoe lake. [1] Temperatura at the surface of the sea or in an aquarium. [2]Interbreeding experiments: parents (S35 – S38), outsprings (S43-S51). [3]Phylogenetically important nucleotide substitutions for specimens S43-S51 present in Table 1.

Table S2. Range of intraspecific and interspecific *p*-distances for 16S and COI gene fragments for *Coryne* species (after Schuchert, 2005) and *Sarsia* species (*S. tubulosa* and *S. lovenii*).

Genus or Species	16S intraspecific	16S interspecific	COI intraspecific	COI interspecific
Sarsia tubulosa	0-1.4%	-	0-1.7%	-
Sarsia lovenii	0-0.7%	-	0-1.7%	-
Sarsia lovenii haplgroup 1/ haplogroup 2	0-0.7%	-	1.2-1.7%	-
Sarsia tubulosa/ *Sarsia lovenii*	-	0.7-2.1%	-	3.9-5.4%
Coryne spp.	0-5.5%	3.7-9.2%	-	-

Schuchert, P. Species boundaries in the hydrozoan genus *Coryne*. *Mol. Phyl. Evol.* **36**, 194–199. http://dx.doi.org/10.1016/j.ympev.2005.03.021 (2005).

Table S3. List of specimens from GenBank used for phylogenetic analyses

Species	Isolate/Voucher Location	Genbank accession numbers			Reference
		COI	16S	ITS	
Cladonema radiatum	DNA370<br>Italy: Island of Elba	MF000495			Schuchert P. Unpublished
Cladonema radiatum	MHNG:INVE-79739<br>France: Roscoff		KP776805		Schuchert P. Unpublished
Coryne eximia	MHNG:INVE-34009 (isolate 182)<br>South Africa: Langebaan	KT981902	AJ878713		Schuchert, 2005a
Coryne eximia	MHNG:INVE-38776<br>Chile	KT981909	KP776758		Schuchert P. Unpublished
Coryne uchidai	MHNG:INVE-49102<br>Japan: Oshoro	KT981912	GQ395320		Schuchert P. Unpublished; Nawrocki et al, 2010
Dipurena halterata	MHNG INVE31741<br>France:Villefranche-sur-mer		AM084261		Schuchert, 2005b
Sarsia apicula	MHNG INVE29806 (isolate Sch285)<br>Canada: British Columbia		GQ395330		Nawrocki et al, 2010
Sarsia bella	Isolate DNA861<br>USA: Friday Harbor	MF000497	MF000538		Schuchert, P. Unpublished
Sarsia lovenii	MHNG INVE48736<br>Norway	KT981910	GQ395329		Schuchert P. Unpublished Nawrocki et al, 2010
Sarsia lovenii	MHNG INVE29592<br>Iceland		AY787876		Cunningham C.W. Unpublished
Sarsia lovenii	MHNG INVE29592<br>Iceland		AJ608796		Schuchert P. Unpublished
Sarsia princeps	Canada: British Columbia		EU876549		Cartwright P., Nawrocki A.M., 2010
Sarsia princeps	Sapr01	GQ120061			Ortman et al., 2010
Sarsia princeps	10PROBE-28265<br>Canada: Manitoba	MG423490			Dewaard J. R. Unpublished
Sarsia princeps	HLC-30031<br>Canada: Nunavut	MG422634			Dewaard J. R Unpublished
Sarsia sp	isolate="MT06847"<br>NorthSea	KC440084			Laakmann S., Holst S., 2013
Sarsia striata	MHNG-INVE-35765 (isolate MHNG-HYD-DNA303)<br>United Kingdom: Scotland		KX355408		Schuchert P. Unpublished
Sarsia striata	MHNG:INVE-35765 (isolate 304)<br>United Kingdom: Scotland	KT981905	GQ395328		Schuchert P. Unpublished; Nawrocki et al, 2010
Sarsia striata	isolate MHNG-HYD-DNA1118<br>Norway		KX355410		Schuchert P. Unpublished
Sarsia striata	isolate MHNG-HYD-DNA305<br>United Kingdom: Scotland		KX355409		Schuchert P. Unpublished

Sarsia tubulosa	MHNG-HYD-DNA1117<br>Norway: Fanafjord		KX355449	Schuchert P.<br>Unpublished
Sarsia tubulosa	? Japan		AB720902	Gotoh et al.<br>Unpublished
Sarsia tubulosa	MHNGINV35763<br>United Kingdom		EU876548	Cartwright P.,<br>Nawrocki A. M.,<br>2010
Sarsia tubulosa	Sch424<br>Norway: Raunefjord		GQ395327	Nawrocki et al,<br>2010
Sarsia tubulosa	? Germany		AY512545	Collins et al., 2005
Sarsia tubulosa	MHNG INVE35763<br>United Kingdom: Scotland		AJ878720	Schuchert P., 2005
Sarsia tubulosa	Satu07<br>?	GQ120063		Ortman et al., 2010
Sarsia tubulosa	Satu06<br>?	GQ120062		Ortman et al., 2010
Sarsia tubulosa	MT06848<br>North Sea	KC440087		Laakmann S.,<br>Holst S., 2013
Sarsiatubulosa	MT03828<br>North Sea	KC440086		Laakmann S.,<br>Holst S., 2013
Sarsia tubulosa	MT03834<br>North Sea	KC440085		Laakmann S.,<br>Holst S., 2013
Sarsia tubulosa	zy03<br>? China	JQ353758		Cheng et al., 2012
Sarsia tubulosa	isolate="FHH03"<br>China	KY767917		Sheng et al., 2018
Sarsia tubulosa	isolate="FHH02"<br>China	KY767916		Sheng et al., 2018
Sarsiatubulosa	isolate="FHH01"<br>China	KY767915		Sheng et al., 2018
Sarsia tubulosa	BIOUG<CAN>:10PROBE-<br>18999<br>Canada	HQ970899		iBOL,<br>Unpublished
Sarsia tubulosa	BIOUG<CAN>:10PROBE-<br>18998<br>Canada	HQ970898		iBOL,<br>Unpublished
Sarsia tubulosa	BIOUG<CAN>:10PROBE-<br>18986<br>Canada	HQ970894		iBOL,<br>Unpublished
Stauridiosarsia cliffordi	MHNG:INVE-78933<br>USA: Friday Harbor	KT981917		Schuchert P<br>Unpublished
Stauridiosarsia producta	MHNG:INVE-48751<br>Norway: Raunefjord	KT981911		Schuchert P<br>Unpublished
Stauridiosarsia producta	MHNG-HYD-DNA431<br>Norway, Raunefjord		KX355421	Schuchert P<br>Unpublished

Cartwright, P., & Nawrocki, A. M. Character evolution in Hydrozoa (phylum Cnidaria). *Integr. Comp. Biol.* 50, 456–472 (2010).

Cheng, F., et al. DNA barcoding of common Medusozoa in northern China based on mtCOI sequence // *Oceanol. limnol. sin.* 43, 451–459 (2012).

Collins, A. G., Winkelmann, S., Hadrys, H., & Schierwater, B. Phylogeny of Capitata and Corynidae (Cnidaria, Hydrozoa) in light of mitochondrial 16S rDNA data. *Zool. Scr.* 34, 91–99 (2005).

Laakmann, S., & Holst, S. Emphasizing the diversity of North Sea hydromedusae by combined morphological and molecular methods. *J. Plankton Res.*, 36, 64–76 (2013).

Nawrocki, A. M., Schuchert, P., & Cartwright, P. Phylogenetics and evolution of Capitata (Cnidaria: Hydrozoa), and the systematics of Corynidae. *Zool. Scr.*, 39, 290–304 (2010).

Ortman, B. D., Bucklin, A., Pagès, F., & Youngbluth, M. DNA Barcoding the Medusozoa using mtCOI. *Deep Sea Res. Part 2 Top. Stud. Oceanogr.*, 57(24-26), 2148–2156 (2010).

Schuchert, P. Species boundaries in the hydrozoan genus *Coryne*. *Mol. Phylogenet. Evol.*, 36(1), 194–199 (2005a).

Schuchert, P. Rediscovery of *Coryne fucicola* (de Filippi, 1866) (Cnidaria: Hydrozoa). *Cah. Biol. Mar.*, 46(3), 305–310 (2005b).

Table S4. Primers and programs used in amplification reactions

Gene fragment	Primer	Sequence/Program	Reference
Internal transcribed spacer region ITS1 and ITS2	SR6R (Small subunit RNA primer)	AAGWAAAAGTCGTAACA AGG	Vilgalys unpubl.
	LR1 (Large subunit RNA primer)	GGTTGGTTTCTTTTCCT	Vilgalys, Hester, 1990
	Program	95 °C for 5 min; followed by 34 cycles of 15 s at 94 °C, 30 s at 52 °C and 60 s at 72°C and then a final extension of 5 min at 72 °C	
Mitochondrial 16S rRNA fragment	16SAR	TCGACTGTTTACCAAAAACA TAGC	Cunningham and Buss 1993
	16SBR	ACGGAATGAACTCAAATCAT GTAAG	
	Program	95 °C for 5 min; followed by 34 cycles of 20 s at 94 °C, 45 s at 50 °C and 120 s at 68°C and then a final extension of 5 min at 68 °C	
Mitochondrial COI fragment	jGLCO1490	TGTAAAACGACGGCCAGT TNTCNACNAAYCAYAARGA YATTGG	Geller et al., 2013
	jGHCO2198	CAGGAAACAGCTATGAC TANACYTCNGGRTGNCCRAA RAAYCA	
	Program	95 °C for 5 min; followed by 34 cycles of 20 s at 95 °C, 60 s at 48 °C and 60 s at 72°C and then a final extension of 5 min at 72 °C	

Cunningham, C. W., Buss, L. W. Molecular evidence for multiple episodes of paedomorphosis in the family Hydractiniidae. *Biochem. Syst. Ecol.*, 21, 57–69 (1993).

Geller, J., Meyer, C., Parker, M., & Hawk, H. Redesign of PCR primers for mitochondrial cytochrome c oxidase subunit I for marine invertebrates and application in all-taxa biotic surveys. *Mol. Ecol. Resour.*, 13, 851–861 (2013).

Vilgalys, R., & Hester, M. Rapid genetic identification and mapping of enzymatically amplified ribosomal DNA from several Cryptococcus species. *J. Bacteriol. Mycol.*, 172, 4238–4246 (1990).

Vilgalys unpubl. https://sites.duke.edu/vilgalyslab/rdna_primers_for_fungi/

Table S5. Crossing scheme of interbreeding experiments for *Sarsia lovenii*

Experiment 1: Male medusa x Female medusoid
Experiment 2: Male medusoid x Female medusa
Positive Control 1: Male medusa x Female medusa
Positive Control 2: Male medusoid x Female medusoid
Negative Control 1: Female medusa x 0
Negative Control 2: Female medusoid x 0
